# Mito‐nuclear discordance at a mimicry color transition zone in bumble bee *Bombus melanopygus*

**DOI:** 10.1002/ece3.8412

**Published:** 2021-12-08

**Authors:** Briana E. Wham, Sarthok Rasique Rahman, Marena Martinez‐Correa, Heather M. Hines

**Affiliations:** ^1^ Department of Entomology The Pennsylvania State University University Park Pennsylvania USA; ^2^ The Pennsylvania State University Libraries University Park Pennsylvania USA; ^3^ Department of Biology The Pennsylvania State University University Park Pennsylvania USA; ^4^ Department of Biological Sciences The University of Alabama Tuscaloosa Alabama USA

**Keywords:** bee, *Bombus*, coloration, hybrid zone, mimicry, mitochondrial–nuclear discordance, speciation

## Abstract

As hybrid zones exhibit selective patterns of gene flow between otherwise distinct lineages, they can be especially valuable for informing processes of microevolution and speciation. The bumble bee, *Bombus melanopygus*, displays two distinct color forms generated by Müllerian mimicry: a northern “Rocky Mountain'’ color form with ferruginous mid‐abdominal segments (*B*. *m*. *melanopygus*) and a southern “Pacific'’ form with black mid‐abdominal segments (*B*. *m*. *edwardsii*). These morphs meet in a mimetic transition zone in northern California and southern Oregon that is more narrow and transitions further west than comimetic bumble bee species. To understand the historical formation of this mimicry zone, we assessed color distribution data for *B*. *melanopygus* from the last 100 years. We then examined gene flow among the color forms in the transition zone by comparing sequences from mitochondrial COI barcode sequences, color‐controlling loci, and the rest of the nuclear genome. These data support two geographically distinct mitochondrial haplogroups aligned to the ancestrally ferruginous and black forms that meet within the color transition zone. This clustering is also supported by the nuclear genome, which, while showing strong admixture across individuals, distinguishes individuals most by their mitochondrial haplotype, followed by geography. These data suggest the two lineages most likely were historically isolated, acquired fixed color differences, and then came into secondary contact with ongoing gene flow. The transition zone, however, exhibits asymmetries: mitochondrial haplotypes transition further south than color pattern, and both transition over shorter distances in the south. This system thus demonstrates alternative patterns of gene flow that occur in contact zones, presenting another example of mito‐nuclear discordance. Discordant gene flow is inferred to most likely be driven by a combination of mimetic selection, dominance effects, and assortative mating.

## Graphical abstract

This study examines the shift in color and genotype (mitochondrial and nuclear) frequencies along a narrow transition zone between mimetic color variants of bumble bee *Bombus melanopygus*. The data support this being a secondary contact zone for two historically distinct populations with different colors, but alternative patterns of gene flow have resulted in different distributions of color alleles from mitochondrial markers. This discordance may be driven by a combination of mimetic selection and asymmetric premating bias, a partial barrier which would place this species along the speciation continuum.

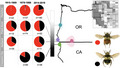

## 1 INTRODUCTION

Debate has surrounded the nature of speciation and whether species are distinct entities or fall more along a continuum (e.g., Mallet, 2008; Twomey et al., 2016). Species delimitation studies often fail to fully define species boundaries in favor of understanding more nebulous patterns of isolation. Geographic hybrid or population contact zones can provide an ideal opportunity to study phylogeographic and speciation processes as they represent regions where both selection and speciation isolation mechanisms are at play (Arias et al., 2008; Barton & Hewitt, 1985; Endler, 1977). The resulting gene or phenotypic frequency clines can inform historical biogeographic processes and the forms of selection maintaining or preventing admixture (Endler, 1977).

Bumble bees (*Bombus*) have a long taxonomic history and we have a relatively thorough knowledge of their species‐level relationships. The boundaries of what is a species, however, remains uncertain in several lineages. Bumble bees exhibit an exceptional diversity of aposematic color patterns that are geographically structured onto numerous Müllerian mimicry complexes worldwide (Ezray et al., 2019; Williams, 2007). Species with ranges that cross more than one mimicry complex often converge onto distinct mimicry patterns as a result of direct selection for specific phenotypic color patterns in different geographic regions (Ezray et al., 2019; Hines & Williams, 2012; Owen & Plowright, 1980; Williams, 2007). The resulting color pattern diversity has generated taxonomic confusion on species composition, which has motivated several studies to assess species status (e.g., Bossert et al., 2016; Duennes et al., 2012; Ghisbain et al., 2020; Hines & Williams, 2012; Koch et al., 2018; Martinet et al., 2018, 2019; Williams et al., 2020). It also has resulted in ample intraspecific polymorphisms that meet in mimicry transition zones (Ezray et al., 2019; Williams, 2007). These zones can be especially informative for interpreting the evolutionary processes driving phenotypic diversity, as selection is likely actively driving the frequency of these color pattern alleles (Owen, 1986).

**FIGURE 1:**
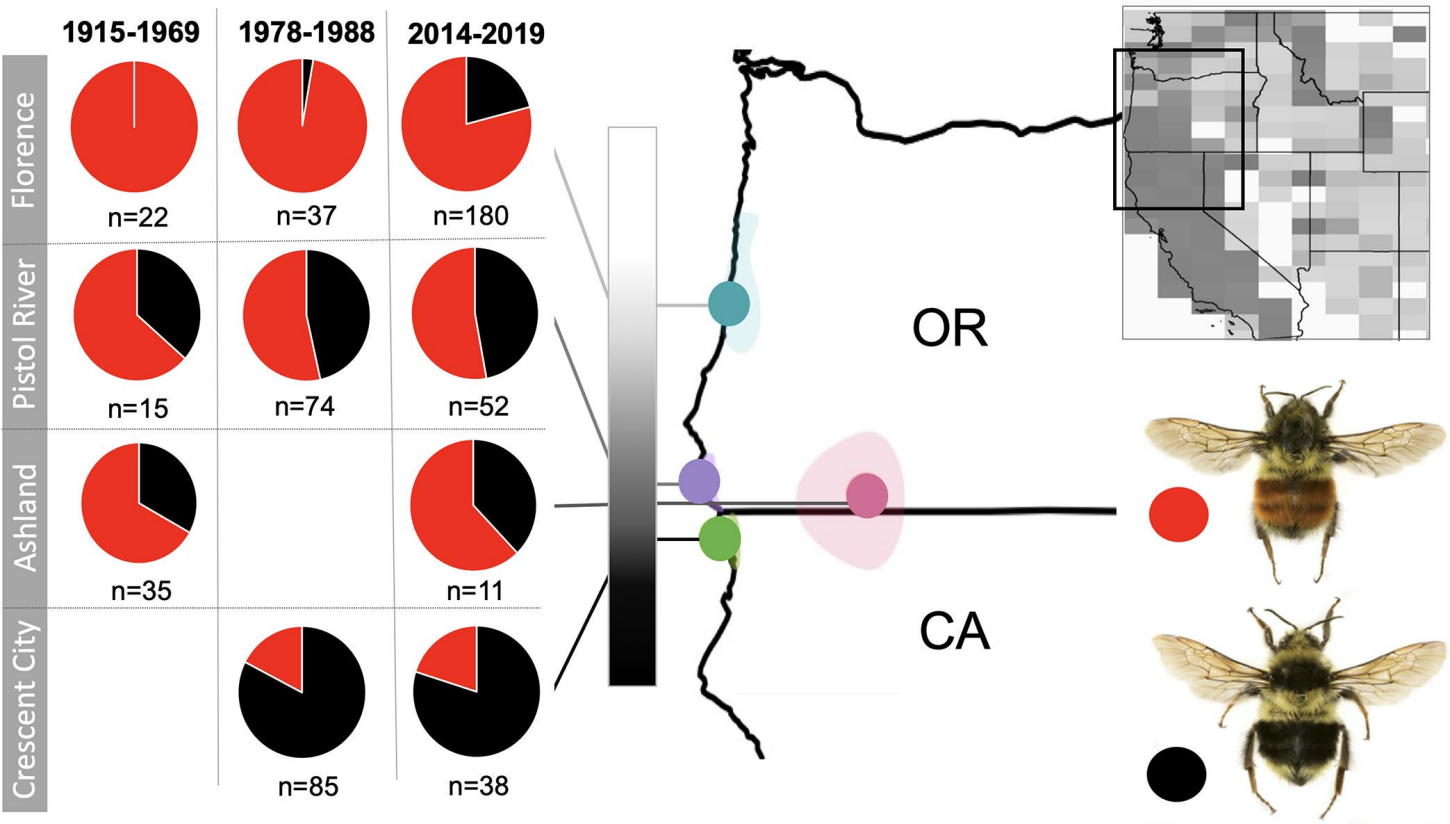
The proportion of ferruginous and black color alleles of *B*. *melanopygus* at four locations within the phenotypic, color hybrid zone (Florence/CapePerpetua, Pistol River, Ashland, and Crescent City) across three time periods (1915–1969, 1978–1988, 2014–2019). Florence represents the northern edge of this hybrid zone, while Crescent City represents the southern edge of this hybrid zone. The gray bar indicates the clinal transition, showing how color transitions more rapidly on the southern edge of the hybrid zone. On the map, opaque colored regions under each colored region depict the ranges of sampling areas where points were selected for each locality. Inset in the upper right corner is a map showing the mimicry transition zone when considering all bumble bee species. This depicts the frequency of color patterns by geographic region where dark gray tends toward the black pattern and lighter gray represents more red patterns in that given geographic region. This map is a grayscale version of the perceptual color average map in Ezray et al., 2019. The black box in this map shows the region of focus at left

*Bombus melanopygus* is a polymorphic species displaying two distinct, geographically separated color forms that adhere to two regional mimicry complexes: a more northern form displaying ferruginous coloration on mid‐abdominal segments in Washington, Oregon, and the Rocky Mountain region, that adheres to the “Rocky Mountain” mimicry complex, and a more southern form that displays black coloration on mid‐abdominal segments in primarily California, that adheres to the “Pacific Coastal” mimicry pattern. These two color forms meet in a transition zone spanning ~300 kilometers (km) in northern California and southern Oregon where their coloration follows a cline (Figure 1) (Ezray et al., 2019; Owen, 1986; Owen & Plowright, 1980; Owen et al., 2010; Thorp et al., 1983). Historically, the ferruginous and the black morphs were denoted as separate species, *B*. *melanopygus* and *B*. *edwardsii* (Franklin, 1912; Milliron, 1971; Thorp et al., 1983). However, population genetic analysis in the hybrid zone using allozyme data, along with laboratory crosses between the morphs, revealed that the color forms are genetically admixed and thus comprise a single species (Owen & Plowright, 1980; Owen et al., 2010). Crosses revealed that the color locus follows simple Mendelian inheritance at a single locus, with ferruginous dominant (Owen & Plowright, 1980), and subsequent works identified this locus to a cis‐regulatory region of a nuclear Hox gene, *Abdominal*‐*B (Abd*‐*B)*, whose differential expression drives this dimorphism (Rahman et al., 2021; Tian et al., 2019). Several other bumble bee species, including *Bombus bifarius* and *Bombus flavifrons*, exhibit the same ferruginous and black polymorphisms of *B*. *melanopygus*, comimicking it across much of its range. However, these species tend to maintain transition zones that are considerably broader and occur further east than *B*. *melanopygus* (Ezray et al., 2019) (Figure 1). It remains to be determined which factors drive the maintenance of a narrower hybrid zone that falls outside the transition zone between the Pacific and the Rocky Mountain mimicry complexes in *B*. *melanopygus*.

Hybrid zones, such as mimicry transition zones, are primarily sustained by a balance between selection and dispersal (Barton & Gale, 1993; Barton & Hewitt, 1989; Thurman et al., 2019) and shifts in this balance can impact transition zone size, structure, location, and ability to move (Barton & Hewitt, 1985). These zones can be expected to narrow in response to selection on hybrids or rare forms and widen in response to gene flow (Thurman et al., 2019). Disruption of the balance between selection and dispersal can cause these zones to move and change over time: as mimicry transition zones are under frequency‐dependent selection, factors which shift color pattern frequency, such as dominance drive, strength of aposematic signals, and range shifts with anthropogenic change, can result in shifting mimicry zones (Barton, 1979; Blum, 2002; Mallet, 1986; Mallet & Barton, 1989).

In this study, we address the factors that influence the mimetic transition zone in *B*. *melanopygus* through studying the phylogeographic history of their dimorphic color forms. We compare the distribution of mitochondrial DNA (mtDNA; *cytochrome oxidase I*) sequences, whole genome nuclear variants, and color pattern phenotypes and alleles across this species’ north–south clinal transition. As gene flow can vary among different genes in the genome due to selection, and selection is likely to take place on color loci because of mimicry, we also assess the history of color loci compared to histories inferred from mitochondrial and nuclear data. These data together reveal the history of this species with respect to their adaptive coloration, how alleles of color and more neutral markers are flowing at the geographic transition zone, and inform how such an unusual transition zone occurs in this species.

## 2 METHODS

### 2.1 Documenting phenotypic color variation across the cline and potential for hybrid zone shifts

To examine the how color patterns shift in *B*. *melanopygus* and whether this transition zone has moved geographically over time, the frequency of black and ferruginous color alleles was inferred across the phenotypic transition zone from specimen data from three time periods: 1915–1969, 1978–1988, and 2014–2019. The historic samples (1915–1969) represent phenotypes collected from museum specimens, including workers and drones (males). A count of ferruginous and black queens collected from 1978–1988 were provided by Robin Owen and were collected by Robin Owen, Chris Plowright, and Monica Geber. These data were not only extracted from Owen (1986) but also included additional unpublished data provided by Robin Owen. The 2014–2019 samples were collected by our research group and include queens, workers, and drones. Data were extracted for collecting events around four localities (depicted in Figure 1): Florence/Cape Perpetua, Pistol River, Ashland, and Crescent City. It has previously been shown that the frequency with which this color allele is found fits with the assumptions of Hardy–Weinberg equilibrium for a single gene with two alleles and ferruginous dominant (Owen & Plowright, 1980). That a single locus is involved was further confirmed by the genomic identification of the color locus (Tian et al., 2019). Thus, the Hardy–Weinberg equation (b^2^ + 2bf + f^2^ = 1), where b^2^ is the proportion of homozygous black individuals, 2bf is the proportion of heterozygous ferruginous individuals, and f^2^ is the proportion of homozygous ferruginous individuals, was utilized to estimate the frequency of black (b) and ferruginous (f) alleles for the diploid queen and worker samples from these regions. As queens and workers can be either heterozygous or homozygous for the color allele, to estimate the frequency of each color allele in females, the frequency of the black allele (b) was first inferred as √(proportion of black homozygous individuals). The frequency of the ferruginous allele (f) was then calculated as 1‐b. Since males are haploid, the frequency of alleles could simply be estimated using the proportion of each color form at a given location. Combined color form frequencies were calculated treating male and female individuals equally (as opposed to treating females as two alleles and males as only one), by combining the number of effective female individuals with each allele (color allele frequency (b or f) * total # of females) and adding this to the number of males that displayed each color form. To test whether there are different proportions inferred over time within each locality, estimated count frequencies of ferruginous and black alleles were compared within each sampling locality across the three time periods (1915–1969, 1975–1988, and 2014–2019) using a chi‐squared test of independence as implemented in the stats package in R (R Core Team, 2017).

### 2.2 Specimen collection for mitochondrial sequencing

We obtained mitochondrial sequences from extracts of 127 *B*. *melanopygus* ferruginous (*n* = 63) color morphs and black (*n* = 64) color morphs collected from throughout most of this species’ range in the western United States. Sampling not only focused primarily on the region where the black form transitions to the ferruginous form (northern California and southern Oregon) but also included samples at the edges of this species’ range in southern California, Washington, Utah, and Wyoming (Figure 1, Table S1). Specimens were either wild‐caught (majority) or reared in the Hines Lab. Wild‐caught individuals were net collected and immediately transferred to 100% ethanol before being stored at −20°C. Reared individuals were removed from colonies founded by wild‐caught queens and directly frozen at −20°C. Individuals sampled included queens, workers, and drones collected in 2003 (1 specimen) and from 2013 to 2018 (Table S1).

### 2.3 DNA extraction and Sanger sequencing of mtDNA

Mid legs, hind legs, or thoracic muscle were removed from each specimen and total genomic DNA was extracted using the E.Z.N.A Tissue DNA Kit (Omega, Bio‐tek) following recommended manufacturer's instructions. A portion of *cytochrome oxidase I (COI)*, a mitochondrial barcode gene, was sequenced for the 127 *B*. *melanopygus* specimens, yielding an approximately 640‐bp fragment (primers LCO1490 and HCO2198) (Folmer et al., 1994). As mtDNA reflects a direct line of maternal inheritance free from recombination and evolves at a rapid rate, it can provide a clearer bifurcating history of both recent and historic geographic events (Avise et al., 1984). PCR was performed using 7.5 µl of Hot Start Taq 2x Master Mix (New England Biolabs), 3.65–5.9 µl of molecular grade water, 0.3 µl of forward and reverse primer, and 1–2 µl of genomic DNA, for a reaction volume of 13.75 µl to 15 µl. The amplification conditions utilized were: 2 min at 94°C, an initial cycle of 30s at 94°C, 40s at 45°C, 1 min at 72°C, then 35 cycles of 30s at 94°C, 40s at 49°C, 1 min at 72°C, and lastly 10 min at 72°C. DNA was purified using ExoSAP‐IT and Sanger sequenced with the forward primer for *COI* (LCO1490) at the Pennsylvania State Genomics Core Facility (University Park, PA).

### 2.4 MtDNA phylogenetic analysis

We performed a Bayesian phylogenetic analysis and haplotype network analysis of mitochondrial *COI* sequences. Sequences were edited manually and aligned in Geneious (v.8.1.9). Single‐nucleotide polymorphism (SNP) variants in alignments were double‐checked against chromatograms and sequence ends were trimmed to a consistent length to reduce the amount of missing data and remove primers. A Bayesian 50% majority rule phylogeny was constructed from 3 runs of 10 million generations each (4 chains, 1000 sample frequency), run in MrBayes 3.2.7a (Ronquist et al., 2012) in CIPRES (Miller et al., 2010). A GTR+I model and flat priors were applied, selected using jModeltest2 v2.1.10 (Darriba et al., 2012) run in CIPRES, and ploidy was designated as haploid. Trees were constructed using a 25% burnin, which was assessed to be sufficient for convergence using the sump function. This tree included the newly obtained sequences for *B*. *melanopygus* as well as outgroup sequences from the closest (Cameron et al., 2007) sister species obtained from the Barcode of Life Datasystem (boldsystems.org), including *B*. *lapponicus* (GBHAP756_14), *B*. *sylvicola* (JSYKA176_10), *B*. *monticola* (GBHAP767_14), *B*. *bimaculatus* (BBHYL248_10), as well as slightly more distant *B*. *impatiens* (HGPPH081_10), which was used to root the tree.

The bifurcating nature of phylogenetic trees does not adhere well to dynamics of populations, as certain haplotypes may represent interior nodes of a tree. Haplotype networks can more accurately represent the evolutionary relationships of intraspecific haplotypes as they allow for multifurcation and reticulation (Posada & Crandall, 2001). Therefore, to further explore the evolutionary dynamics among individuals of *B*. *melanopygus*, a haplotype network was created in the program Population Analysis with Reticulate Trees (PopArt (7.1); Leigh & Bryant, 2015) using the TCS method.

### 2.5 Genome sequencing data

#### 2.5.1 Sampling

To compare the geographic distribution of mitochondrial haplotypes in the transition zone to geographic structure in the nuclear genome, we performed phylogenetic and population genetic analysis of polymorphic sites across the whole genome, excluding allelic variants in the color locus, from 18 previously published genomic samples spanning the transition zone (Tian et al., 2019) and 2 newly sequenced southern mtDNA haplotype (see mitochondrial results) samples that span the range of this haplotype. The resulting sampling included 10 individuals of each color form, with northern mtDNA haplotypes (*n* = 17) including individuals across the hybrid zone as well as one individual from Washington, USA, and the southern mtDNA haplotypes (*n* = 3) including an individual from the northernmost extent of the southern haplotype near the northern edge of the hybrid zone, a sample from the middle of the hybrid zone, and a sample far south of the hybrid zone in the Sierra Nevada of California, USA (Table S1).

#### 2.5.2 Genome sequencing, alignment, and variant calling

For the newly sequenced samples, DNA was extracted from muscle and leg tissues as for *COI*, libraries were prepared for sequencing using an Illumina Nextera DNA library construction kit, and libraries were sequenced 150 bp paired end using the Illumina NextSeq 550 sequencer at the Pennsylvania State Genomics Core Facility (University Park, PA). Quality assessment, read trimming, and alignment of genomic reads to the *B*. *impatiens* RefSeq genome assembly (BIMP_2.0, GCA_000188095.2, NCBI) were conducted using methods described in Tian et al. (2019). Multisample variant calling was conducted utilizing all 20 genomes using GATK v.3.6–0 (McKenna et al., 2010), which generated a raw dataset of 6,269,539 SNPs.

#### 2.5.3 Variant filtering and NUMT removal

For the population genetic analysis, we sought to retain only high‐quality SNPs across the genome. To filter SNPs, we applied initial quality filtering retaining only SNPs which were biallelic, have a minimum depth of 3 and minimum genotype quality greater than or equal to 20. To exclude potentially contaminated, misassembled contigs, or fragments of non‐nuclear genomes, we discarded SNPs from contigs shorter than 20 kb. We then discarded SNPs with any missing data across samples, which generated a dataset of 1,462,409 SNPs.

As we wanted to make sure our nuclear signal was only nuclear in origin, we excluded SNPs from mtDNA or nuclear mitochondrial DNA (NUMT) regions from our genomic sequences using a multitiered approach. We first discarded all SNPs which were within genomic regions annotated as “mitochondria” and “mitochondria‐like” (*n* = 666) in the *B*. *impatiens* genome assembly. We then used sequences from the mitochondrial genomes of two closely related species, *B*. *pratorum* (GenBank Accession: KT164684.1, KT164685.1, KT164686.1) and *B*. *ignitus* (GenBank Accession: DQ870926.1), to conduct a BLAST search against the *B*. *impatiens* reference genome and excluded SNPs from regions of match. Finally, we examined SNPs that were fully fixed (*n* = 407) between southern black (*n* = 3) versus rest of the genomic samples (*n* = 17) through genome‐wide association analysis (GWAS) implemented in PLINK v.1.90 (Chang et al., 2015) and removed any of the remaining SNPs from this set that were mitochondrial in origin as identified using the blastn program of NCBI BLAST+2.6.0 command line tools (Camacho et al., 2009). All variant filtering procedures were conducted using VCFtools v. 0.1.1 (Danecek et al., 2011).

### 2.6 Population genetic analysis using genome data

To confirm that we were examining the population history outside of the history of the color locus, we excluded the SNPs (*n* = 68) from the previously identified ~18 Kb color interval (Tian et al., 2019). The final filtered dataset of SNPs (*n* = 1,452,678) from outside the color locus were utilized in three methods for inferring patterns of population structure: a neighbor‐joining (NJ) tree, principal component analysis (PCA), and parse non‐negative matrix factorization (sNMF) analysis. A neighbor‐joining phylogeny was produced in TASSEL 5.2.29 (Bradbury et al., 2007) using the Analysis >Cladogram and Clustering method: neighbor‐joining option, and closely related *B*. *impatiens* was used to root the tree.

For PCA and sNMF analysis, highly linked SNPs were removed from the dataset using bcftools v. 1.10.2 (Li, 2011) “prune” plugin using the following parameters (‐m 0.5 ‐w 5 kb; prevents more than *r* ^2^ > 0.5 in a window of 5 kb) to generate a 293,776 SNP dataset. Calculations of principal components were conducted in PLINK v.1.90 (Chang et al., 2015) and plotted in R “ggplot2” (Wickham, 2016). sNMF analyses were implemented using the LEA package (Frichot & François, 2015). Ten independent runs were conducted from a range of K‐values (K = 1 to K = 10) and the optimal K‐value representing the number of ancestral populations was inferred from the evaluation of minimum cross‐entropy calculation.

We also examined whether the SNPs that are fixed between southern and northern mtDNA haplotype individuals (described above) might be clustered in certain places in the genome. If genomic clustering occurs this could be indicative of genomic regions that limit gene flow, and which may thus act as speciation loci. We analyzed distances between SNPs and the number of SNPs per contig and then examined Manhattan plots for clustered regions. We also compared the clustering of fixed SNPs between the haplotypes of identified NUMTs and non‐NUMT SNPs.

### 2.7 Color locus analysis

Several of the specimens (89 *B*. *melanopygus*; 47 ferruginous and 42 black) sequenced for COI were also sequenced for the most associated region of the color locus, a noncoding sequence in the region of Abd‐B (Tian et al., 2019). This 1,464‐bp region has shown perfect fixation of genotype to color phenotype in the fixed SNPs identified in this region by genome sequencing (*n* = 21; 10 fixed SNPs and 1 fixed indel) across all sequenced individuals thus far (*n* = 133; Tian et al., 2019). Thus, this region should have a low impact of recombination with opposing color forms and best reveal the history of the color locus. To compare divergences and historical patterns within *B*. *melanopygus* with that of other relatives with similar monomorphic or polymorphic coloration, a set of closely related species (Cameron et al., 2007) were also sequenced for this locus, including single representatives of both black and ferruginous forms of species that vary in the same segments as *B*. *melanopygus*: *B*. *impatiens* (black; sequence extracted from the *B*. *impatiens* RefSeq genome assembly (BIMP_2.0, GCA_000188095.2, NCBI)), *B*. *ternarius* (ferruginous), *B*. *vancouverensis nearcticus* (ferruginous and black forms), *B*. *ephippiatus* (ferruginous and black forms), *B*. *huntii* (ferruginous), *B*. *vosnesenskii* (black), *B*. *sylvicola* (ferruginous and black forms), and *B*. *bimaculatus* (black). Specimens were amplified with primers 1F and 1R (Tian et al., 2019) and followed a PCR cycle similar to *COI* except for an annealing temperature of 52°C for 38 cycles, and samples were sequenced with both primers, necessary to obtain the full sequence with some overlap. Sequences were analyzed as for *COI*, using both Bayesian approaches and TCS haplotype networks. The Bayesian phylogeny was constructed with all nucleotide sites (model determined in jModeltest: GTR+G; run as for COI except 5,000,000 generations and ploidy=diploid) as well as a separate partition for gap‐coded characters (*n* = 11 characters) modeled under a coding=variable binary model. The haplotype network also included gap characters but excluded individuals that were heterozygous for haplotypes (*n* = 81 of 89 melanopygus included). Outgroups for haplotype networks included one individual each of the three closest relatives: *B*. *bimaculatus*, *B*. *sylvicola* (ferruginous form), and *B*. *sylvicola* (black form).

In addition to obtaining a phylogeny from the most associated region, we also extracted SNPs from a larger region of the color locus (18 Kb; 294 SNPs)—the same region excluded from the genomic analysis—from the above analyzed genome sequence samples. We then performed a NJ phylogenetic analysis following the method outlined above on all SNPs that were present in at least 80% of individuals.

### 2.8 Mitochondrial, color, and nuclear clinal structure

To assess whether clinal patterns for mtDNA haplotypes, color phenotypes, and nuclear genome structure differed, we compared modeled clines along the north–south transition by fitting equilibrium cline models using the hzar package in R (Derryberry et al., 2014). This package implements the Metropolis‐Hastings Markov chain Monte Carlo (MCMC) algorithm. Models were fit for each data type separately and the clinal center and width was estimated for each. Clinal analyses only included individuals that are located along the north–south cline between southern California and Washington, thus, samples that were sequenced to assess mtDNA haplotype identity in Wyoming and Utah were removed. The mtDNA haplotype cline model used both wild and reared individuals sequenced in this study (*n* = 125). A phenotypic cline model was fit using the color pattern data from our study samples plus the museum records from Ezray et al. (2019) that fell within the north–south cline range extent of the specimens sequenced in this study (*n* = 1694). The nuclear cline used the genomic samples and their estimated percent population assignment from the population assignment plot (*n* = 20). Because the hzar package was primarily designed to build clines based on abundant population samples within discrete localities and our data were not collected in this manner, we designated each individual specimen as a sampling locality so that the likelihood calculation did not vary by sample size. We acknowledge that this may present problems with modeling from our empirical data. Specimens were analyzed along a 1D linear transect estimated as the latitudinal distance from the southernmost locality using the distHaversine function in the geosphere package (Hijmans, 2017) in R v3.6.2. Each cline was constructed using the MCMC operator default parameters. Three cline models (I: scaling = “fixed” [minimum and maximum values are fixed to the minimum and maximum values of the data], tails = “none” [model includes no exponential tails]; II: scaling = “free” [minimum and maximum values are estimated], tails = “none”; III: scaling = “free”, tails = “both” [model includes two tails which are fitted separately]) (Derryberry et al., 2014; Derryberry & Derryberry, 2015) were run and model performance was compared using the Akaike information criterion corrected for small sample size (AICc).

To further explore if the observed distribution of color phenotype and mtDNA haplotype combinations diverges from null expectations and is representative of mitochondrial–nuclear (mito‐nuclear) discordance, chi‐squared goodness‐of‐fit tests were conducted using the stats package in R v3.6.2 (R Core Team, 2017) for individuals sampled for the mtDNA haplotype within the hybrid zone. This included first comparing if the distribution of haplotypes is proportional to the distribution of color alleles. Considering the black form is derived from the southern mtDNA haplotype and the ferruginous form from the northern mtDNA haplotype, if gene flow is the same between color and mitochondria then the frequency of mitochondrial haplotypes in the hybrid zone should match the frequency of color alleles. For this analysis, expected frequencies for each mtDNA haplotype were the percentage of color alleles. As bumble bee males are haploid, we inferred the color allele directly from the color phenotype. Additionally, for female specimens sequenced for the color locus, color alleles are known. Thus, using all male samples and only the females with known color alleles (Table S1), we calculated the frequency of color alleles present within the hybrid zone for each haplotype. Second, we inferred whether mtDNA haplotypes have the same color distribution by assessing whether each mtDNA haplotype differed in their color frequency relative to the total percent of each color in the hybrid zone. As most individuals in the hybrid zone are the northern mtDNA haplotype, one would expect that the distribution of colors from northern mtDNA haplotypes does not differ in distribution from the overall color frequencies, therefore, this statistic is most valuable for assessing whether the distribution of color forms in the southern mtDNA haplotype is different from that in the northern mtDNA haplotype. To further visualize discordance in our sampled specimens, a color phenotype cline model was fit using color pattern data only from the sequenced specimens from this study (*n* = 125) using the methods described above and compared visually to the mtDNA haplotype cline.

### 2.9 Wolbachia analysis

*Wolbachia* infections—symbiotic vertically transmitted bacterial infections largely confined to the reproductive tract—have been implicated to be a potential cause of mito‐nuclear discordance among populations, as it can generate sex‐based reproductive barriers that cause differential gene flow in maternal compared to paternal lines and lead to rapid selective sweeps of mitochondrial haplotypes (Toews & Brelsford, 2012). *Wolbachia* has been found to infect >20% of arthropod species tested (Pascar & Chandler, 2018) and has previously been found to occur in many lineages of Hymenoptera, including in bumble bees (Li et al., 2011). To infer whether the presence of *Wolbachia* might explain patterns of discordance in our molecular data, we performed two different approaches. First, we used *Wolbachia* primers to detect whether *Wolbachia* amplified via PCR in gut tissues of bumble bees of each color form. Then we screened for the presence of *Wolbachia* transcripts in a *de novo* assembly of an embryonic transcriptome of *B*. *melanopygus*.

#### 2.9.1 PCR methods

Sterile technique was utilized to remove all gut tissue except the cuticle from a total of 21 samples, 9 individuals displayed the southern mtDNA haplotype (all black) and 12 individuals displayed the northern mtDNA haplotype (5 black and 7 ferruginous). Total genomic DNA was then extracted from gut tissue using the E.Z.N.A Tissue DNA kit (Omega, Bio‐Tek) as above, with the exception that tissue was more thoroughly homogenized using metal beads (*n* = 3 beads per bee) on low for 35 seconds in an Omni Bead Ruptor. PCR was performed as above except with 2 min at 95°C, then 35 cycles of 30s at 95°C, 30s at 49°C, 1 min 72°C, and then 5 min at 72°C, and using Wsp 81F and Wsp 691R primers, two primers previously used to assess for *Wolbachia* across insects (Pascar & Chandler, 2018; Zha et al., 2014), including ants (Wenseleers et al., 1998) and bumble bees (Gerth et al., 2011). Bees were also amplified with housekeeping gene elongation factor 1a (EF‐1a) F2 copy primers for bumble bees (Cameron et al., 2007) to ensure DNA quality was sufficient to obtain amplification. A comparative PCR was run at higher, 52°C, and lower, 46°C, temperatures than the original 49°C amplification to determine if varying temperatures provided different results. A subsequent PCR was performed on eight samples, four of northern mtDNA haplotype and four of southern mtDNA haplotype, with DNA volume increased by 1 μl to see if this would instead yield products.

Extracted *Wolbachia* DNA from cultured mosquito cells (age 2) infected with wMel strain of *Wolbachia* were used as a positive control for all assays. All PCR samples were run through standard gel electrophoresis and *Wolbachia* was considered present if bands were detected.

#### 2.9.2 Transcriptomic methods

As PCR‐based approaches could potentially miss *Wolbachia* if strains deviate in sequence too much from primers, to further investigate the possibility of *Wolbachia* infection in *B*. *melanopygus*, we assembled a de novo transcriptome assembly of *B*. *melanopygu*s embryos. As *Wolbachia* is passed down from parent to offspring, if *Wolbachia* is present, it should be found within embryos. RNA extraction was performed on a pooled embryo sample extracted from brood from a single black form *B*. *melanopygus* colony. The embryos were homogenized for 35 s on low in the Trizol buffer in the Omni Bead Ruptor, with 4 metal beads, followed by extraction using standard protocols in the Directzol RNA Miniprep Plus kit (Zymo Research). Sufficient RNA quality and quantity were confirmed using an Agilent Bioanalyzer, followed by preparation for Illumina sequencing using a TruSeq Stranded mRNA Kit (Illumina). The sample was sequenced 75‐bp paired‐end at the Pennsylvania State Genomics Core Facility (University Park, PA) using the Illumina NextSeq 550. Initial quality assessment of the generated raw reads (20.417 million pairs) was conducted in FastQC v0.11.5 (Andrews, 2010), followed by removal of adapters, retention of high‐quality reads with a minimum length of 36 bp in Trimmomatic v. 0.39 (Bolger et al., 2014) using the following parameters (SLIDINGWINDOW:4:30 ILLUMINACLIP:adapters.fa:2:30:5 MINLEN:36 LEADING:3 TRAILING:3). De novo transcriptome assembly was performed on trimmed paired‐end reads (14.697 million pairs) using Trinity assembler v. 2.8.5. The resulting transcriptome was 59.829 megabases in size, with 40731 transcripts, and a contig N50 of 2838 bases.

To search for *Wolbachia* in the assembled transcriptome, we compiled *Wolbachia* transcript sequences (*n* = 44) from arthropods from Pascar and Chandler (2018) and queried these against all assembled embryo transcripts utilizing “tblastx” in NCBI BLAST+ (Camacho et al., 2009), retaining hits with an e‐value <0.00001.

## 3 RESULTS

### 3.1 Assessment of the color cline and potential for allelic shifts

Our analysis of the frequency of black and ferruginous color alleles across three time periods (1915–1969, 1978–1988, and 2014–2019) showed, similar to previous studies sampling across the 1978–1988 time period (Owen & Plowright, 1980; Owen et al., 2010), a rather smooth geographic color cline from northern California to southern Oregon. This cline more rapidly transitions from black to ferruginous at the southern end of the transition zone, with slower transitioning to fully ferruginous at the northern end (Figure 1). There was no significant change over these three time periods in the frequency of ferruginous and black alleles across three of the four sites (Ashland: *p* = 1, Crescent City: *p* = .9166, and Pistol River: *p* = .7505) (Figure 1); however, a potential northern shift of the black allele over time is inferred at the most northern extent of the hybrid zone in Florence, OR (*p* = .002).

### 3.2 MtDNA haplotype variability: haplotype network and phylogeny

**FIGURE 2:**
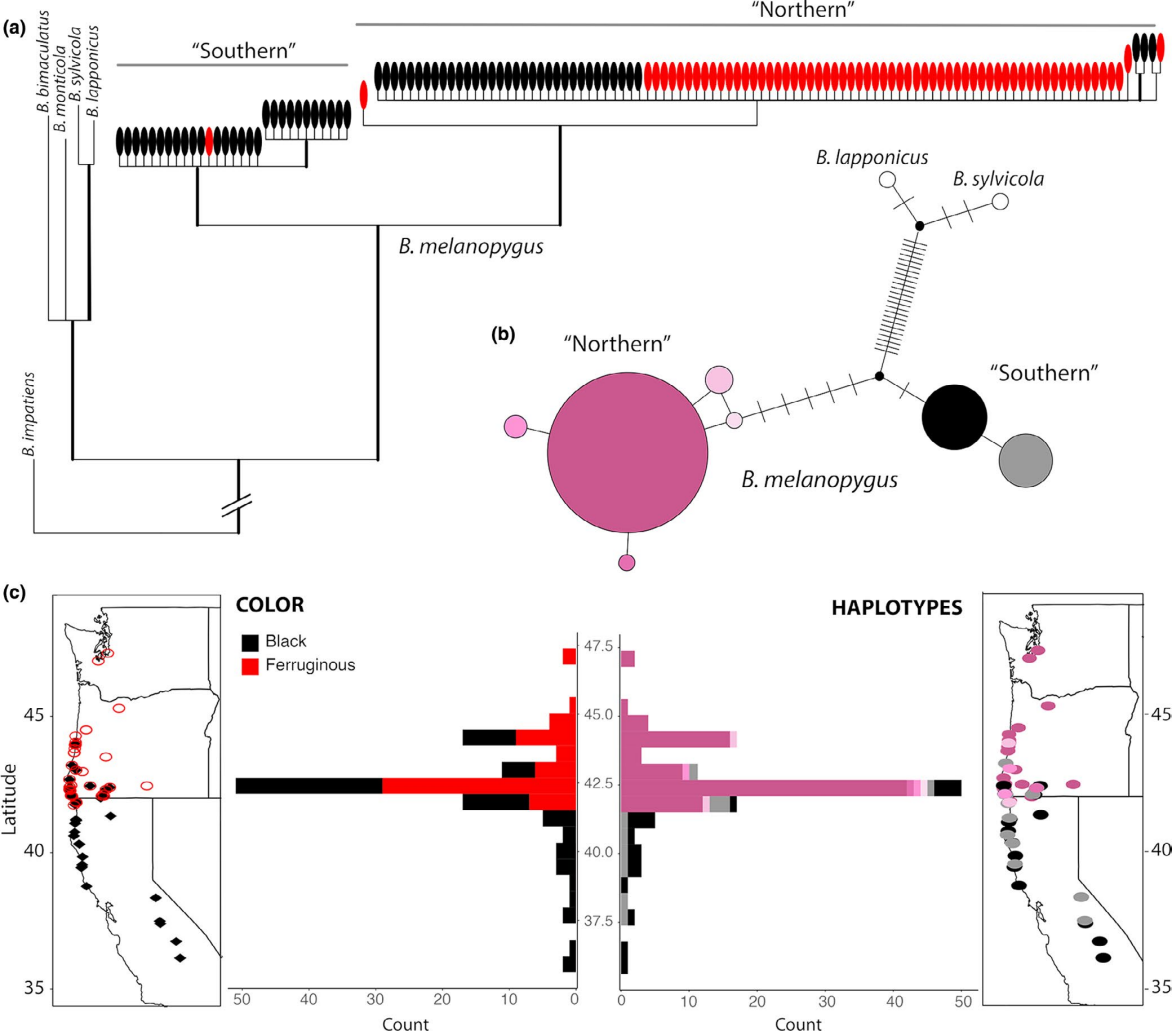
Relationships and distribution of COI haplotypes in *B*. *melanopygus* relative to color patterns. (a) COI Bayesian phylogenetic tree of *B*. *melanopygus* displaying the divergence of mitochondrial haplotypes into northern and southern mtDNA haplotypes. Ovals depict specimen phenotypic color form (ferruginous vs. black) on abdominal tergites 2 and 3. (b) Haplotype network of COI *B*. *melanopygus* sequences. The size of the circles are proportional to the number of specimens with that haplotype, branches without dashes represent a single nucleotide change and dashes represent single step nucleotide changes not represented by any haplotypes, and the colors of the circles are used to depict the geographic distribution of haplotypes in part C. (c) Geographic distributions of samples included in this study on a map and in a histogram by latitude, with color forms (left; ferruginous [open red circles] vs. black [black diamonds]) and COI haplotypes (right; colors match the haplotype colors from the haplotype network in Part B) indicated. Two samples from Wyoming and Utah, respectively, are included in the haplotype network (northern main haplotype) but are not graphed in Part C to focus on the coastal latitudinal cline

Both Bayesian phylogenetic analysis and haplotype network analysis of mitochondrial COI sequences revealed strong support for two divergent mitochondrial clades representing northern and southern haplogroups along the west coast of the United States (Figure 2). Across all samples, there were 13 variable sites with 12 being parsimony informative, with most of the variation between these two clades, which differ by eight nucleotides (Figure 2). This represents ~1.6% sequence divergence, which equates to an approximate divergence just over 1 million years ago assuming an evolutionary rate for *COI* of 1.5%/million years (Quek et al., 2004). The two haplotypes form a monophyletic group relative to much more divergent outgroup species (e.g., on average 5.8% divergence between *B*. *melanopygus* and sister lineages *B*. *lapponicus*, *B*. *monticola*, *B*. *sylvicola*, *B*. *bimaculatus*, and *B*. *impatiens*).

Phylogenetic patterns (Figure 2) show that each haplogroup is geographically restricted, exhibiting a northern haplotype and a southern haplotype that overlap only where the color forms meet in southern Oregon and northern California. Bees are fixed for the northern mtDNA haplotypes at localities in Utah, Wyoming, Washington, and northern Oregon, while bees are fixed for the southern mtDNA haplotypes at localities in California south of Crescent City, CA. In the transition zone, both northern and southern mtDNA haplotypes can be observed, although northern haplotypes are considerably more abundant (Figure 2). Both ferruginous and black phenotypic color forms were detected with the northern mtDNA haplotype, whereas only black phenotypic color forms were detected with the southern mtDNA haplotype, with the exception of a single worker with a ferruginous phenotype carrying the southern mtDNA haplotype obtained from a reared, wild‐caught queen (Figure 2, Table S1).

### 3.3 Structure in the nuclear genome

**FIGURE 3:**
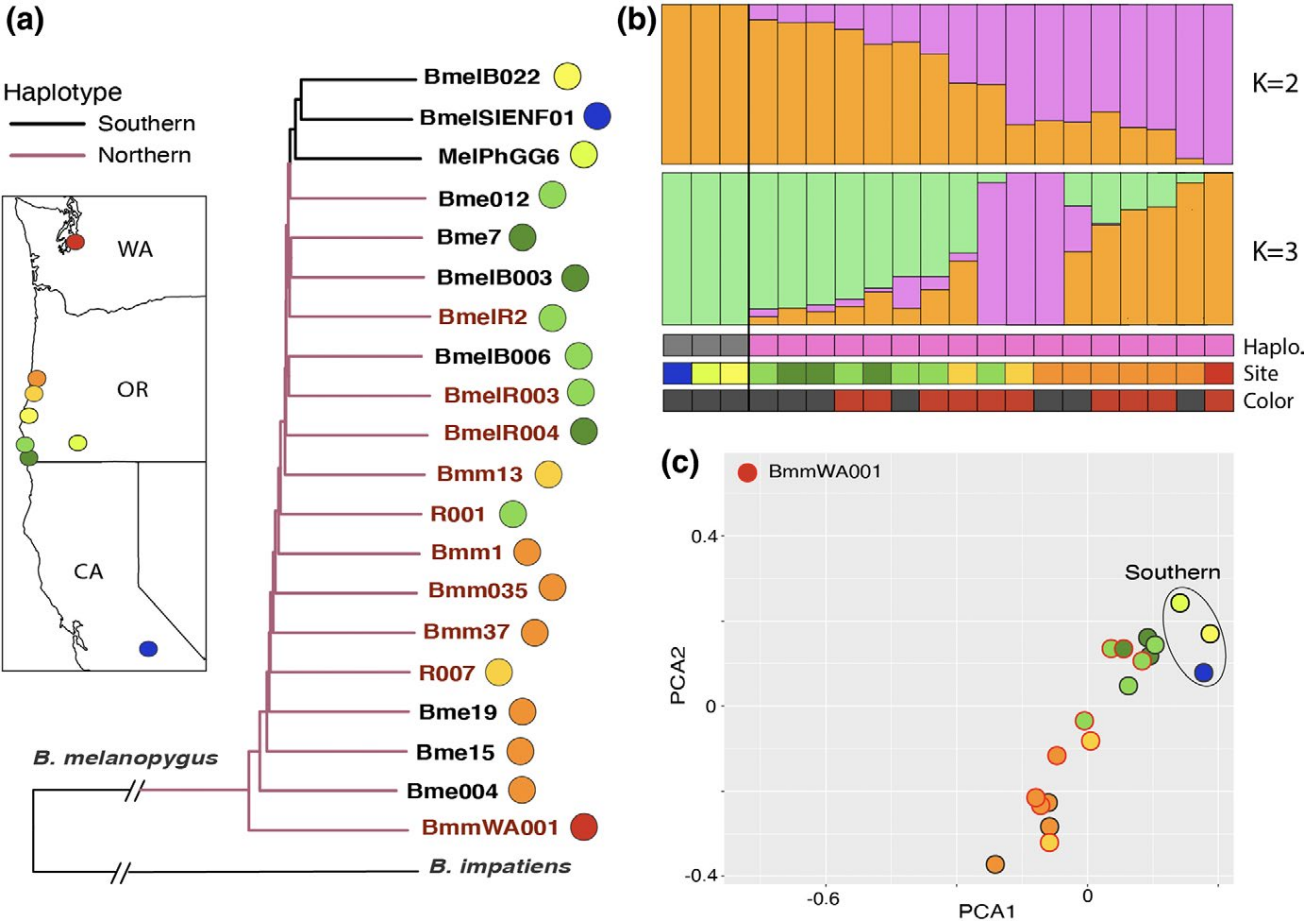
Patterns of variation across genomic samples spanning the *B*. *melanopygus* hybrid zone. (a) NJ phylogeny inferred from genomic SNPs excluding the color interval, including only SNPs with no missing data. Branch color represents haplotype, name color represents color phenotype, and circles represent geographic location as outlined on the map. (b) Admixture/STRUCTURE sNMF analysis results for genomic data from Part A. K = 1 was the most likely and K = 2 and K = 3 were progressively the next most likely (inferred from minimum cross‐entropy calculation plot, Figure S2). Each bar represents a genomic sample and the proportion assigned to each respective population. Color phenotypes (red, black), geographic location (colors match map in Part A), and mitochondrial haplotypes (northern, southern) of each individual are indicated. (c) Plot summarizing variation across the first two principal component axes of individuals with genomic data from Part A. Circles colored by locality and outline colored by color form (ferruginous vs. black), and the three southern haplotype individuals are indicated

**FIGURE 4:**
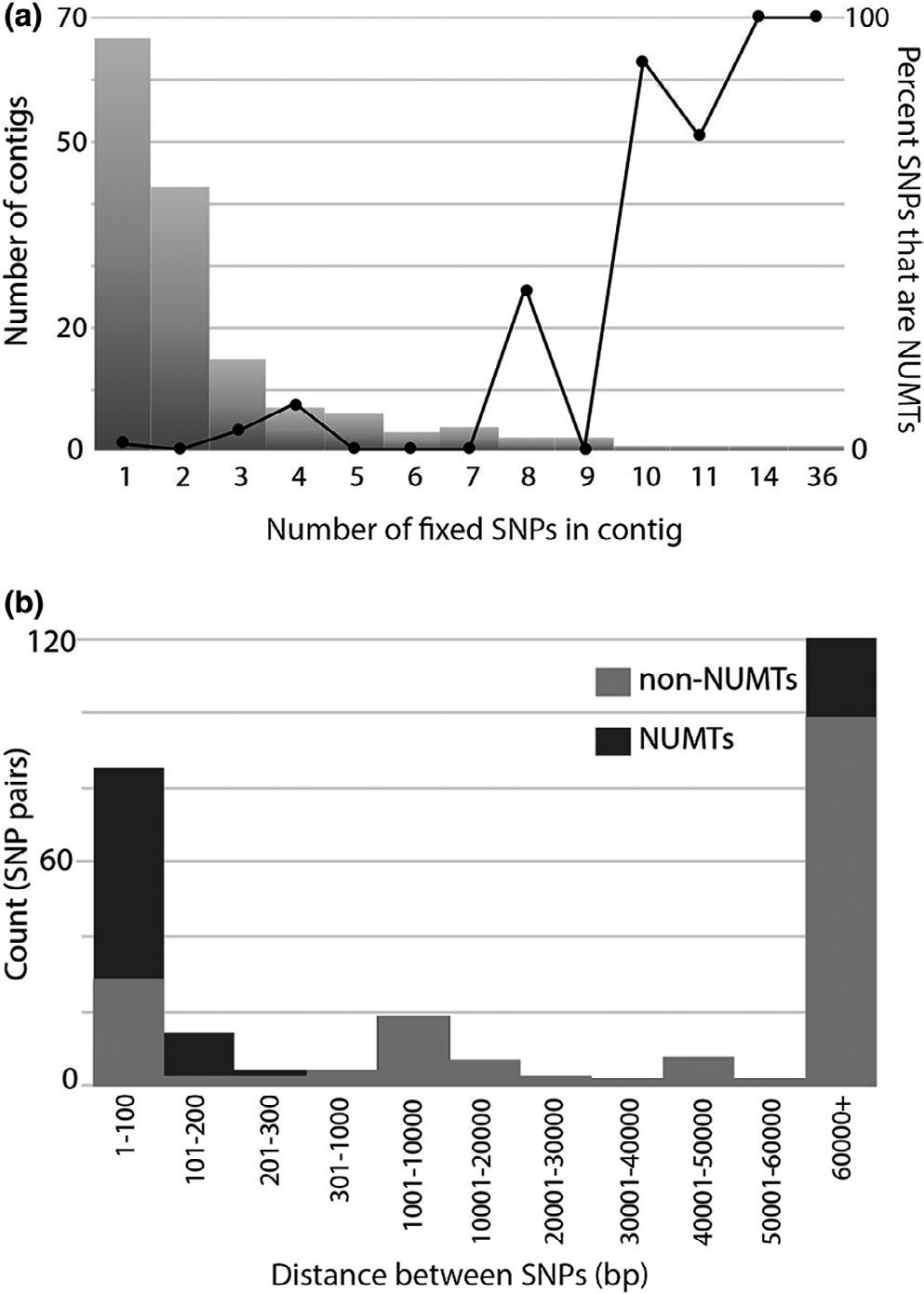
Distribution across genomic contigs of SNPs that are fixed between the northern and southern haplotype genomes, including distinction of whether these SNPs are mitochondrial gene copies contained in the nuclear genome (NUMTs). (a) The bar plot representation shows a histogram of the number of fixed SNPs per contig, revealing that most contigs only have a few fixed SNPs. The line plot shows the percent of all SNPs in contigs with that number that are NUMTs. This shows that the contigs which have many SNPs tend to contain NUMTs. (b) Genomic distance (bp) between fixed SNP pairs for both NUMTs and non‐NUMTs. The majority of SNPs in close proximity (clusters of fixed sites) are NUMTs

The nuclear genomic data from 20 *B*. *melanopygus* individuals spanning the hybrid zone revealed a distinct cluster of the three individuals with the southern mitochondrial haplotype separate from the rest (evidenced by phylogenetic, sNMF analysis, and PCA (Figure 3)). These are united even though they are sampled across the range of the hybrid zone, suggesting they carry a shared history in both mitochondrial and nuclear markers. However, this divergence is not strikingly different, suggesting considerable gene flow is occurring between the groups. When we examined the SNPs that support this relationship, although there are many more group‐associated SNPs, of the total 4,107,952 SNPs, 328 non‐NUMT SNPs are completely fixed between the mitochondrial haplotypes. These SNPs were scattered across the genome with little clustering, thus no one particular region is supporting this pattern (Figure 4). This provides support against a specific nuclear locus of divergence that might promote species barriers. Prior to excluding NUMTs, genomic clusters of SNPs were found that were centered on NUMTs (i.e., most SNPs in close proximity were NUMTs, Figure 4; 79 fixed SNPs in NUMTs). This signal could result from recent mitochondrial to nuclear transfer or misalignment of more abundant mitochondrial DNA reads to nuclear DNA. These results suggest that failure to remove NUMTs from genomic data can bias mitochondrial and nuclear comparisons.

The genomic data also show structuring of individuals along a north–south geographic cline, with the tree rooted in northern populations and southern populations appearing progressively more derived in the tree (Figure 3). While sNMF analysis has the highest support for a single admixed population, not far behind in likelihood is a two‐population designation. The southern mtDNA haplotype individuals are most distinct, but aside from this, these two populations separate individuals primarily by geographic location along the cline (Figure 3). Genomic data in the hybrid zone do not cluster by color form and adding a third population does not demarcate individuals by color above other types of population clustering (Figure 3), suggesting color has different patterns of gene flow from the rest of the genome. There is higher genetic variation between samples in the northern part of the hybrid zone, as evidenced by greater divergences in the phylogeny and separation in the PCA plot (Figure 3). Greater similarity at the southern end of the hybrid zone is also supported by the Washington sample and southern California sample being separated from the hybrid zone by a similar geographic distance, but the Washington sample having a much higher degree of distinction from the other samples (Figure 3).

### 3.4 Color locus evolution

**FIGURE 5:**
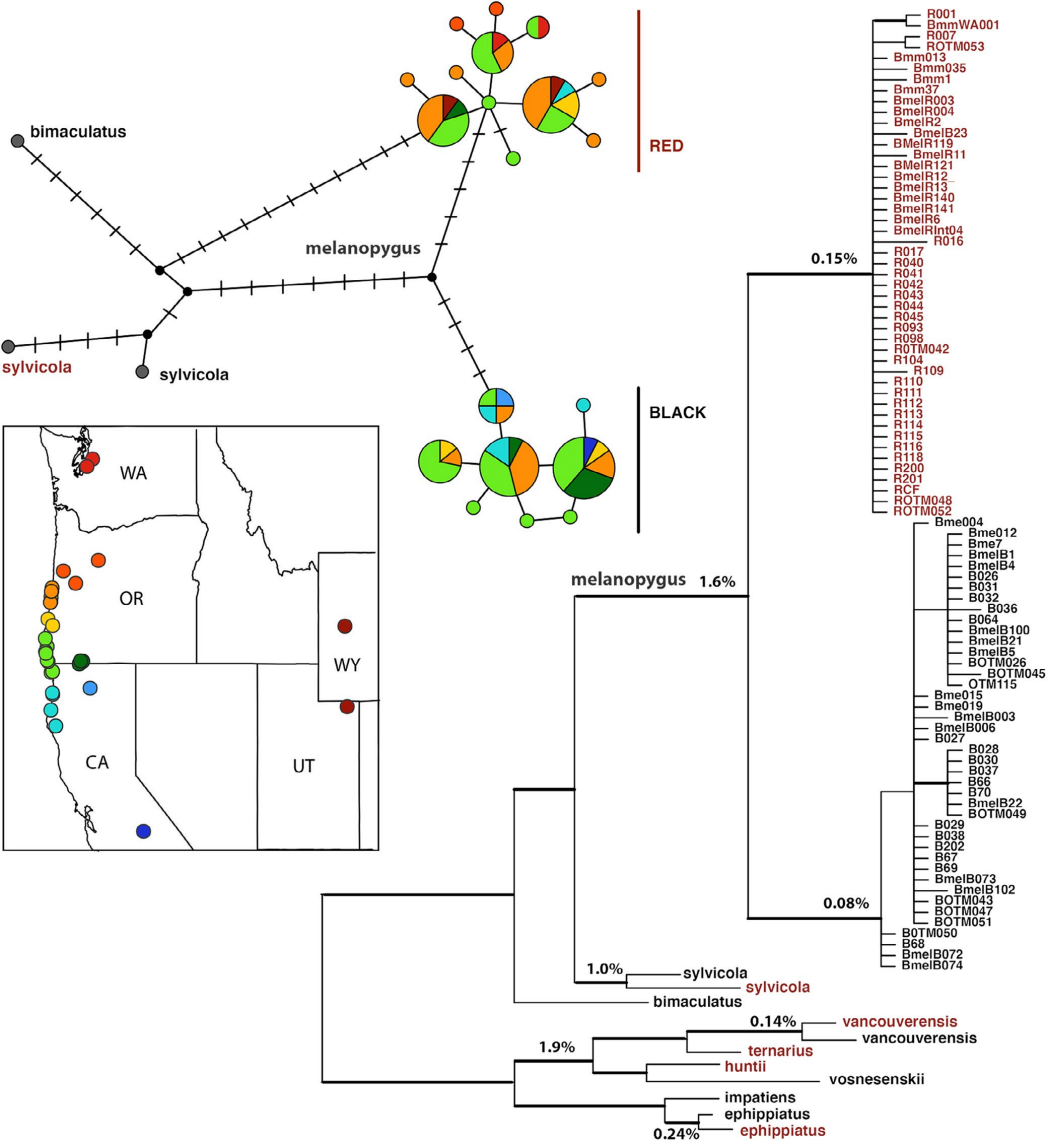
Bayesian phylogeny and haplotype network inferred from the fixed component of the color locus (1464 bp, cis‐regulatory region 3’ of *Abd*‐*B*). The Bayesian phylogeny (right) depicts color forms (red and black color) of *B*. *melanopygus* and outgroups. Thick branches are supported with Bayesian posterior probabilities >0.95. Percentages on branches are the average percent pairwise sequence divergence for that node. The haplotype network (top) of these data depicts the geographic distribution of these samples, colored relative to locations depicted in the map. The number of changes is depicted by lines, with no line for 1 change, and circle size depicts the number of individuals comprising each haplotype

The Bayesian phylogeny and haplotype network of color locus sequences revealed this locus to be monophyletic for all *B*. *melanopygus*, lending support to color variation being independently evolved in *B*. *melanopygus*, and not acquired through introgression from similar forms in other species or sorting of ancestral variation. Within this clade, there is substantial divergence within *B*. *melanopygus* that sorts by color forms, a result that is not unexpected given that this is the region of complete fixation and may contain the color‐generating SNPs. The divergence between color forms at this locus exceeds the divergence typical across more distantly related species (Figure 5), suggesting that selection is driving sequence change. Most black and ferruginous outgroup species pairs, which in each case, unlike *B*. *melanopygus*, come from geographically disjunct populations, vary comparatively little, although *B*. *sylvicola* shows more considerable divergence by color form at this locus. Ferruginous and black *B*. *melanopygus* forms each have acquired a similar number of changes from the ancestor and exhibit similarly low levels of variation within each form, thus the results do not implicate one color form as the ancestor of the other. The haplotype network shows that within each color form the haplotypes are admixed across the sampled geographic range, supporting broad gene flow within forms at this locus. The phylogeny constructed from the most associated region of the color locus, a noncoding sequence in the region of Abd‐B (18 kb), from genome sequence samples revealed a similar phylogenetic pattern to the narrower (<2 kb) color genetics region, although, unlike the narrower interval, there is some clustering by geography within the ferruginous color form (Figure S1).

### 3.5 MtDNA haplotype, color phenotype, and nuclear biogeographic clinal structure

**FIGURE 6:**
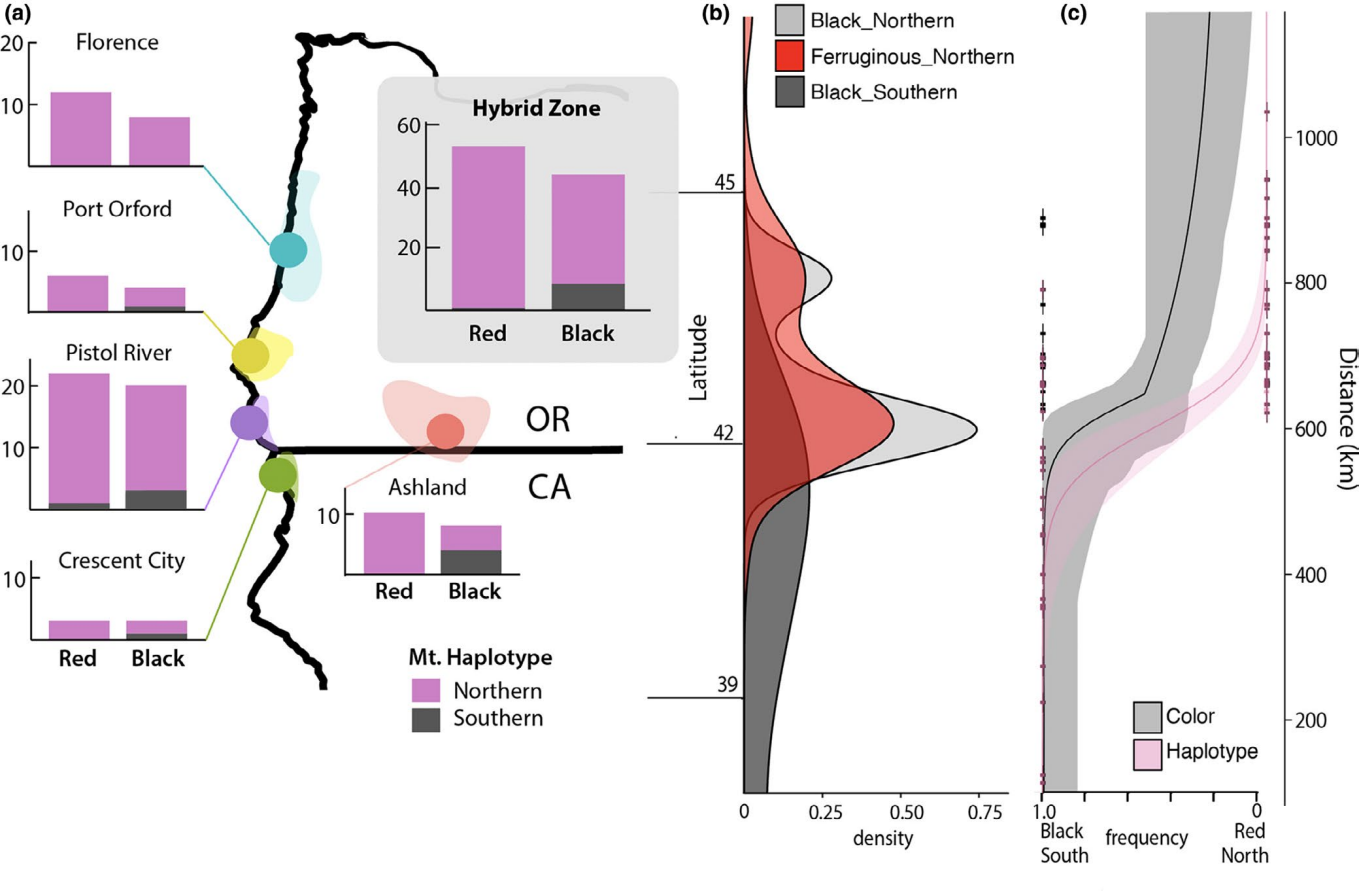
Distribution of mitochondrial haplotypes and color phenotypes in barcoded individuals across the *B*. *melanopygus* transition zone. (a) Distribution of mitochondrial haplotypes (northern, southern) across barcoded individuals of each color form (ferruginous = red, black) across five regions of the color transition zone. These specimens are used for Chi‐square analysis of discordance. Borders around each zone indicate the range of points for that region. In gray is the overall distribution of mtDNA haplotypes in the hybrid zone across each color form, showing that the northern haplotype dominates in the hybrid zone regardless of color form, but that more black forms carry the southern haplotype. (b) Density curves from data used for this analysis for each of the three observed combinations of color and mtDNA haplotype. Only one individual was ferruginous with a southern mtDNA haplotype and given that this is insufficient for modeling distribution, it is not shown. (c) Estimated clines for both color phenotype and mtDNA haplotype based on only the samples sequenced for COI in this study. Distance on the *y*‐axis is latitudinal distance in kilometers from the southernmost specimen and match distances in Figure 7. All three plots are scaled to the same latitudinal axis, thus the latitudes shown between (a) and (b) apply to (a–c)

**FIGURE 7:**
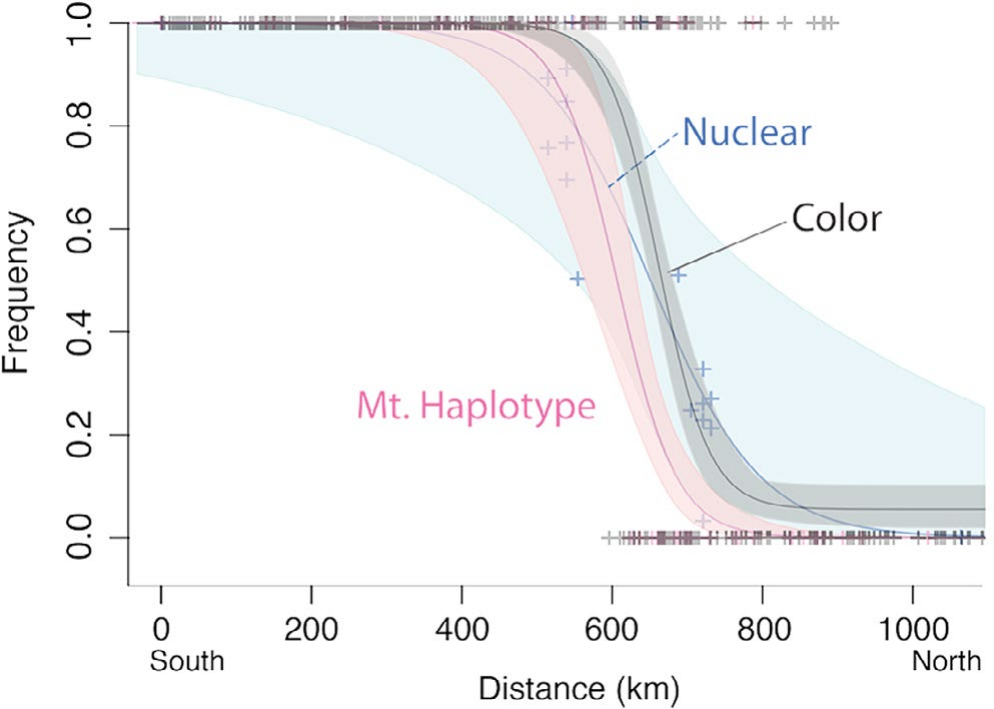
Comparisons of the three major clines across the *B*. *melanopygus* transition zone: nuclear genome, mtDNA haplotype, and color phenotype. Unlike Figure 6, here the color phenotype cline is based on color phenotypes gathered from samples sequenced for COI in this study as well as museum specimens. The mtDNA haplotype cline is inferred from samples sequenced for COI in this study. The nuclear genome cline is inferred from the population assignment plot in Figure 3b from whole genome data. Distance on the *x*‐axis is latitudinal distance in kilometers from the southernmost specimen. Curves are modeled from discrete ferruginous‐black color and southern‐northern mtDNA haplotypes, and continuous data for nuclear data, with each specimen treated as a separate locality for all three models

Model I (scaling = fixed, tails = none) was selected as the best fit model for the MtDNA haplotype and nuclear clines, whereas model II (scaling = free, tails = none) was selected as the best fit model for the two‐color phenotype clines (study samples only and study samples + museum samples) (Table S2). When we compared modeled clines along the north–south transition for mtDNA haplotypes, color phenotypes, and the nuclear genome (Figures 6 and 7; Table S2), clinal variation in mitochondrial markers (COI) and color phenotype showed discordance geographically, highlighting the occurrence of mito‐nuclear discordance in this system. There was an approximately 54.7‐km shift between the center of the color phenotype (661.6 km; cline center range: 648.5–674.1 km) and mtDNA haplotype cline (607.0 km; cline center range: 558.3–635.0 km). The differences between mtDNA haplotype and color phenotype clines in Figure 7 would be even more pronounced (the color phenotype cline would be further right‐shifted) if we had inferred clines for color alleles rather than phenotypes given that ferruginous phenotypes are dominant.

Chi‐square goodness of fit tests, which were run using color alleles instead of color phenotypes, support the northern mtDNA haplotype being disproportionately represented in the hybrid zone compared to the expectations based on color allele frequencies (*p* = 6.618e‐14) (Figure 6a). The distribution of ferruginous and black color alleles in the hybrid zone across individuals expressing the northern mtDNA haplotype did not differ from overall frequencies of these color alleles in the hybrid zone (*p* = .3711), but there was a significant bias among southern mtDNA haplotypes, which exhibit more black color alleles (*p* = .004231) (Figure 6). Distribution curves also show imbalanced rates of change, with an abrupt decline in the northern haplotype at the southern edge of the transition zone, in contrast to a more gradual cline in color (Figures 6b,c and 7).

Although inferences are tentative for nuclear genome samples given more limited data, the observed center of the nuclear genome cline (Figure 7, based on Figure 3b) was shifted slightly south of the color phenotype cline, but not to the southern extent of the mtDNA haplotype cline. The center of the nuclear cline (650.6 km; cline center range: 551.4 to 801.8 km) was shifted 11 km south of the center of the color cline and 43.7 km north of the center of the mtDNA haplotype cline. The hzar cline models inferred clinal widths (inverse of maximum slope) of 134.3 km for color phenotypes (study samples + museum samples), 157.6 km for mtDNA haplotypes, and 292 km for the nuclear genome (Table S2).

### 3.6 Wolbachia incidence

The PCRs across conditions and specimens did not yield any sign of *Wolbachia* in these bees, while yielding strong bands in our *Wolbachia*‐positive control sample and in *EF*‐*1a* control genes run on the bee DNA. The transcriptomic approach to detect *Wolbachia* infection did not yield any positive results either. Although several (*n* = 21) BLAST hits passed an e‐value threshold of <0.00001, these were short sized (18–131 bp), with low similarity (25.19–48.33%) and all fell within the bee‐specific heat shock protein 60A and 10‐kDa heat shock proteins, which share a low level of similarity with the *groE Wolbachia* heat shock proteins (Masui et al., 1997).

## 4 DISCUSSION

### 4.1 Phylogeographic history of *B. melanopygus*

The contemporary distribution of mtDNA haplotype diversity, nuclear genome signal, and geographic patterning in *B*. *melanopygus* together most likely support a population history including a period of isolation starting approximately 1 million years ago, after which color phenotypes likely became fixed into a northern, ferruginous form and southern, black form, in adherence to the local mimicry complexes. This would have been followed by a more recent secondary contact of these haplogroups to build the current color transition zone. *COI* data lend support for these two populations diverging from an ancestral haplotype that is now extinct in sampled populations; however, nuclear genome data root the tree in more northern populations, with more similarity of northern populations with the outgroup, and the data support northern populations harboring more genetic diversity than southern populations. This would suggest that the southern population may be derived from more northern populations prior to their isolation, which makes sense given that this is a southwestern extreme of a much broader western geographic distribution and that bumble bees in general are cold adapted and historically have moved southward in the Nearctic from Palearctic regions (Hines, 2008). Given that the color locus supports color variation arising within the *B*. *melanopygus* clade, this suggests the black form most likely evolved from an ancestral ferruginous form, as has been previously suggested from genetic diversity data at the color locus (Tian et al., 2019). Other population patterns, however, such as differences in historical population size and regional patterns of gene flow, could explain the reduced diversity among individuals of southern haplotypes.

The presence of a genetic transition zone in the absence of a geographic barrier to dispersal likely reflects historical biogeographic events (Yang & Kenagy, 2009). Glacial recession has caused there to be hotspots of hybrid, contact and phylogeographic break zones (Remington, 1968; Swenson & Howard, 2005). The location of the hybrid zone between the northern and the southern haplotype of *B*. *melanopygus* is a hotspot for mtDNA divergences across organisms (Miller et al., 2006; Soltis et al., 1997; Yang & Kenagy, 2009), created by the separation of two glacial refugia, one in the Pacific Northwest and one in California. The divergence in *B*. *melanopygus* is likely to have been driven by these glaciation events.

Although it has been argued that distinct mtDNA lineages can be explained by parapatry involving low dispersal and low population size of neighboring populations (Irwin, 2002), in this case, parapatry seems unlikely. Bumble bees have high dispersal rates and thus tend to have low geographic population structure across broad ranges (Lozier et al., 2011). Additionally, the two lineages in *B*. *melanopygus* are in a commonly recognized geographic hybrid zone, have color phenotype features that accompany the mtDNA haplotype differences, and outside the hybrid zone, the mtDNA haplotypes and color phenotypes are distinct, mostly invariant, and fixed, suggesting a history of isolation with substantial gene flow within the respective clades.

### 4.2 Patterns of gene flow across a mimicry transition zone

Although the transition zone is in a similar region for mtDNA haplotype and color phenotype, the clinal analysis for mtDNA haplotypes, color phenotype, and nuclear genome data, along with the asymmetric distribution of color forms along each clade of the phylogenetic tree, indicates discordance between mtDNA haplotype and color phenotype/nuclear clines. In the color transition zone, the southern mtDNA haplotype is much more rare and appears to infiltrate the hybrid zone only in black individuals, whereas the northern mtDNA haplotype occurs readily between both ferruginous and black individuals, suggesting a differential history of gene flow of mitochondrial haplotypes relative to color alleles in areas of admixture.

Mitochondria and color show differences in rates of gene flow in the contact zone. Color transitions more gradually on the northern end away from the contact zone than on the southern end where it transitions more abruptly, whereas the mitochondrial transition zone shifts abruptly near the southern point of contact. Black individuals bearing the southern haplotype show a gradual cline northward, whereas ferruginous and black individuals with the northern mtDNA haplotype end abruptly at the southern edge where ferruginous color ceases to occur. Thus, if we consider the mitochondrial transition zone as the point of contact, ferruginous alleles tend to be restricted in moving southward more than black alleles are restricted in moving northward.

### 4.3 Explanations for discordance

While, typically, there is concordance between mtDNA and nuclear DNA patterns (Zink & Barrowclough, 2008), this is not always the case (Egger et al., 2007; Funk & Omland, 2003; Roca et al., 2005; Toews & Brelsford, 2012; Yang & Kenagy, 2009). Many explanations exist to explain the occurrence of such mito‐nuclear discordance, such as demographic differences between the markers, adaptive introgression of mtDNA, sex‐bias and mate preferences, hybrid zone shifts, *Wolbachia* infections, and human introductions (Gompert et al., 2008; Hudson & Turelli, 2003; Pavlova et al., 2013; Toews & Brelsford, 2012). Below we explore some possible explanations for the transition zone patterns observed in *B*. *melanopygus*.

#### 4.3.1 Assortative mating

**FIGURE 8:**
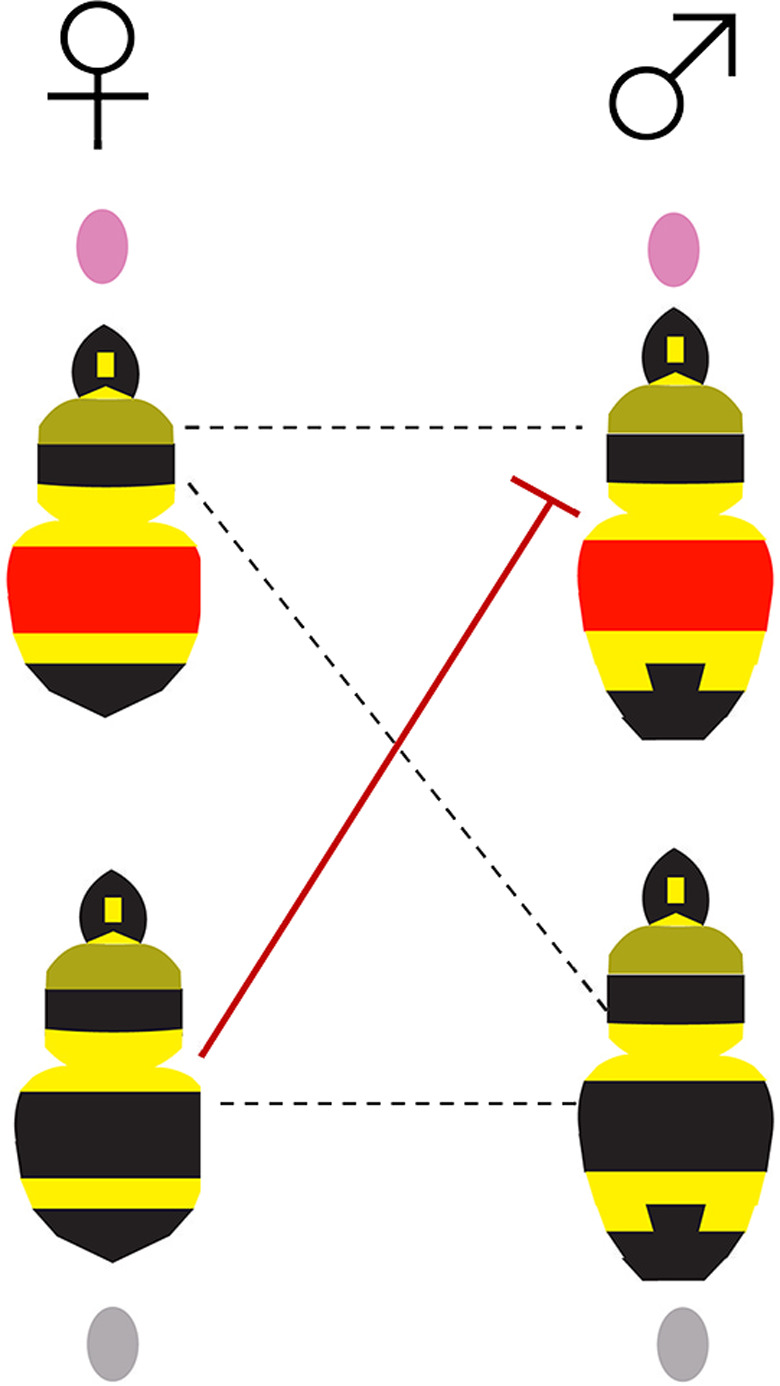
Depiction of a possible scenario of an asymmetric sex bias leading to partial introgression and mito‐nuclear discordance. Under this hypothesized scenario, in the wild, black morph queens carrying the southern haplotype will selectively choose not to mate with males displaying the ferruginous color pattern and possessing the northern haplotype. Alternatively, potentially these two do mate, but do not produce viable offspring

One possible explanation for the imbalance of haplotypes by color form is female‐biased mate preference between haplogroups. As mtDNA is maternally inherited, female‐specific mate biases, especially those that relate to properties generated by mitochondria themselves, could potentially drive differences in gene flow in mitochondria from that seen in nuclear markers (Ballard & Whitlock, 2004). If queens displaying the ancestrally black southern mtDNA haplotype infrequently mate or fail to generate viable offspring with drones displaying the ancestrally ferruginous northern mtDNA haplotype, but queens displaying the ferruginous northern mtDNA haplotype will readily mate with drones displaying the black southern mtDNA haplotype, the ferruginous northern mtDNA haplotype will generate black offspring with the northern mtDNA haplotype in subsequent generations, while the black southern mtDNA haplotype will fail to generate ferruginous offspring with the southern mtDNA haplotype (Figure 8). As ferruginous is dominant to black (Owen & Plowright, 1980), the fairly even mix of black and ferruginous individuals detected with the northern mtDNA haplotypes could suggest that several generations of mixing likely have taken place among individuals with this mtDNA haplotype. A mating bias could explain the skew in rate of change in the transition zone as it would limit gene flow more in the southern part of the range as only dispersal of northern females can allow southern movement of alleles, as dispersing males would not successfully mate. While Wolbachia can be the driving factor responsible for such sex‐specific biases, in this case, we did not find evidence of Wolbachia across either lineage.

#### 4.3.2 Mimicry‐driven selection and the interacting effects of dominance

While mating bias could explain the observed asymmetries in gene flow, a combination of mimetic selection and allelic dominance could explain these patterns as well. Our molecular examination of the color locus provides evidence for selection occurring at this locus, as there are multiple linked SNPs distinguishing color forms, substantial genetic change in this locus compared to sister lineages, and loss of ancestral sequences, suggesting sweeps of these loci. Previous analyses of selection at this locus using genetic differentiation (*F*_ST_), per site nucleotide diversity (π), and linkage disequilibrium (LD) decay on genomic data indicate signatures of recent selection that are stronger in the black color morphs (Tian et al., 2019). The color transition zone of *B*. *melanopygus* is located further west than the typical transition zone between Pacific Coastal and Rocky Mountains mimicry forms, making it the only species with a full ferruginous phenotype in coastal Oregon and S. Washington, where a predominantly black color form is normally found (Ezray et al., 2019). Plotting species abundances by month using preserved specimen data extracted from the Global Biodiversity Information Facility (GBIF) records of common bumble bee species in the *B*. *melanopygus* transition zone (Figure S2), reveals that *B*. *vosnesenskii*—a black form species—dominates year round, supporting selection favoring the black form. While individually not greater in numbers than *B*. *vosnesenskii*, the mixed phenotype *B*. *melanopygus* combined with *B*. *mixtus*, which has an in‐between color form (Ezray et al., 2019), are more abundant in the transition zone in early spring, which could weaken selection at this time of year against the ferruginous form (Figure S2). Overall, by principles of mimetic frequency‐dependent selection, ferruginous forms should be more heavily selected against. This directional selection would thus favor the flow of black color alleles northward and prevent gene flow southward of the ferruginous alleles, a pattern observed in our data.

One factor that historically has been argued to drive shifts in mimicry zones is dominance drive. Under this hypothetical scenario, the color allele that is dominant would acquire higher phenotypic frequency relative to allele frequency, which can cause the enlarging over time of the mimicry zones for dominant phenotypes through frequency‐dependent selection (Mallet, 1986). If we consider the mitochondrial cline as the contact point, our data would suggest against this as it disfavors the movement of the dominant ferruginous alleles southward. Rather, as a result of their dominance, any individual harboring a ferruginous allele south of the contact zone should be selected against. Black alleles, however, can occur without manifesting black phenotypes in the heterozygous state, thus enabling hidden increases in black allele frequency and infiltration of black alleles northward. Reduced selection against the black form in the north could help support black alleles when they become homozygous. The combination of selection and dominance would thus support the observed more rapid shift in phenotypes in the south but would not explain as well as assortative mating why the southern haplotype occurs almost exclusively in black individuals.

Although this could help explain asymmetries, the black form cannot be that strongly favored considering that it has yet to replace the ferruginous forms in northern Oregon. The width of mimicry transition zones in bumble bees are broader than those found in *Heliconius* butterflies (Mallet et al., 1998), which could be a result of weaker mimetic selection and less precise mimicry in bumble bees in general (Ezray et al., 2019). While the maintenance of the transition zone suggests selection is taking place, the width of the transition zone in *B*. *melanopygus* relative to other hybrid zones is consistent with a scenario of weaker selection under equilibrium cline theory (Barton, 1979; Barton & Gale, 1993; Bazykin, 1969; Mallet et al., 1990). Previous research examining this color locus cline suggested that with a potentially overestimated dispersal range of 5–10 km, a selection pressure of 1% or less was enough to sustain the cline (Owen, 1986). The narrower transition zone in *B*. *melanopygus* than in other bumble bee species could be due to a combination of a recent contact after isolation and the more simple single‐locus Mendelian inheritance of their coloration, which would facilitate adaptive change (Ezray et al., 2019).

The observed northward shift of black alleles in the northern part of the transition zone in recent times could be demonstrating the favoring of these alleles, or perhaps short‐term stochasticity. Shifts in transition zones are not uncommon. In *Heliconius* butterflies, transition zones have been found to shift over short periods of time. For instance, a mimicry transition zone in Panama between *H*. *erato petiverana* and *H*. *erato hydara* was found to have shifted approximately 47 km west over 17 years potentially due to dominance drive coupled with deforestation (Blum, 2002; Mallet, 1986) and continued to move, albeit at a slower rate, in the 15 years since (Thurman et al., 2019). In contrast, a mimicry transition zone in northern Peru between *H*. *erato* and *H*. *melpomene* has remained stable from 1985 to 2012 likely due to consistent selection on the color pattern loci and the location being a region of low population density (Rosser et al., 2014). More data are needed across time to assess the stability of these shifting patterns in *B*. *melanopygus*.

#### 4.3.3 Ecological constraints

While black color alleles could be selected to move northward after the point of contact, selection driven by extrinsic environmental factors could shape distribution of mtDNA haplotypes, as mtDNA plays a vital role in metabolism and energy dynamics (Ballard & Whitlock, 2004). Acquired ecological adaptations to different habitats may help to maintain isolation upon secondary contact (Barton & Hewitt, 1985; Mallet et al., 1998) and evidence in *Drosophila* shows that mtDNA haplotypes can possess temperature‐dependent fitness (Matsuura et al., 1993). Temperature norms range widely across the distribution of *B*. *melanopygus*, including more drastic fluctuations of temperature in the extent of northern mtDNA haplotype than is found in the extent of the southern mtDNA haplotype. If a particular mtDNA haplotype is better adapted to the climatic conditions in the transition zone, introgression of the mtDNA may be imbalanced (Ballard & Whitlock, 2004). In this case, the southern mtDNA haplotype may be maladaptive in the hybrid zone and thus may maintain more rarity overall than the northern mtDNA haplotype, whereas the northern mtDNA haplotype could be maladaptive in the region of the southern haplotype.

## 5 CONCLUSIONS

Given previous research showing lack of allozyme population structure in the transition zone, we did not expect to see much mtDNA variation across *B*. *melanopygus*. We were surprised to find that mtDNA markers are consistent with a history of isolation followed by secondary contact in this lineage that has generated a dynamic contact zone. Through studying this zone both at mitochondrial and nuclear levels, we have revealed asymmetries in gene flow, whereby the center of the color transition zone is displaced from the mitochondrial transition zone and shows different rates of gene flow. One explanation for this is assortative mating, in which case *B*. *melanopygus* would not be a simple admixed species, but rather may fall somewhere along the speciation continuum (Mallet, 2008). Alternatively or in addition, this pattern could be generated from a combination of mimetic color selection and the impacts of selection on dominant alleles. These data suggest there may be ongoing shifts in frequencies in this zone in need of further inquiry.

## CONFLICT OF INTEREST

None declared.

## AUTHOR CONTRIBUTIONS

**Briana E. Wham:** Conceptualization (equal); Data curation (equal); Formal analysis (lead); Investigation (lead); Visualization (equal); Writing – original draft (lead); Writing – review & editing (supporting). **Sarthok Rasique Rahman:** Conceptualization (supporting); Data curation (equal); Formal analysis (supporting); Investigation (supporting); Visualization (supporting); Writing – original draft (supporting); Writing – review & editing (supporting). **Marena Martinez‐Correa:** Formal analysis (supporting); Investigation (supporting); Writing – original draft (supporting); Writing – review & editing (supporting). **Heather M. Hines:** Conceptualization (equal); Data curation (supporting); Formal analysis (supporting); Funding acquisition (lead); Investigation (supporting); Project administration (lead); Visualization (equal); Writing – original draft (supporting); Writing – review & editing (lead).

## Supporting information

Figure S1

Figure S2

Table S1

Table S2

## Data Availability

The datasets generated and/or analyzed during the current study are available in Scholarsphere repository (https://doi.org/10.26207/g5yy‐4c33). Sequence data are available on Genbank with Genbank references provided in Table S1 and alignment files are available in the Scholarsphere repository. Raw sequencing reads for the transcriptome are available from NCBI SRA Accession SRP344889 and the transcriptome assembly is available in Scholarsphere. Raw genome sequencing reads are available from NCBI BioProject PRJNA526235 and PRJNA778415 and the raw SNP dataset in vcf format is available at Scholarsphere.

## References

[ece38412-bib-0001] Andrews, S. (2010). FastQC: A quality control tool for high throughput sequence data. Retrieved from http://www.bioinformatics.babraham.ac.uk/projects/fastqc

[ece38412-bib-0002] Arias, C. F. , Munoz, A. G. , Jiggins, C. D. , Mavarez, J. , Bermingham, E. , & Linares, M. (2008). A hybrid zone provides evidence for incipient ecological speciation in *Heliconius* butterflies. Molecular Ecology, 17(21), 4699–4712. 18828780 10.1111/j.1365-294X.2008.03934.x

[ece38412-bib-0003] Avise, J. C. , Bermingham, E. , Kessler, L. G. , & Saunders, N. C. (1984). Characterization of mitochondrial DNA variability in a hybrid swarm between subspecies of bluegill sunfish (*Lepomis macrochirus*). Evolution, 38, 931–941. 28555798 10.1111/j.1558-5646.1984.tb00364.x

[ece38412-bib-0004] Ballard, J. W. , & Whitlock, M. C. (2004). The incomplete natural history of mitochondria. Molecular Ecology, 13(4), 729–744. 10.1046/j.1365-294X.2003.02063.x 15012752

[ece38412-bib-0005] Barton, N. H. (1979). The dynamics of hybrid zones. Heredity, 43(3), 341–359. 10.1038/hdy.1979.87

[ece38412-bib-0006] Barton, N. H. , & Gale, K. S. (1993). Genetic analysis of hybrid zones. In R. G. Harrison (Ed.), Hybrid zones and the evolutionary process (pp. 13–45). Oxford University Press.

[ece38412-bib-0007] Barton, N. H. , & Hewitt, G. M. (1985). Analysis of hybrid zones. Annual Review of Ecology and Systematics, 16(1), 113–148. 10.1146/annurev.es.16.110185.000553

[ece38412-bib-0008] Barton, N. H. , & Hewitt, G. M. (1989). Adaptation, speciation and hybrid zones. Nature, 341(6242), 497–503. 10.1038/341497a0 2677747

[ece38412-bib-0009] Bazykin, A. D. (1969). Hypothetical mechanism of speciaton. Evolution, 23(4), 685–687. 10.2307/2406862 28562864

[ece38412-bib-0010] Blum, M. J. (2002). Rapid movement of a Heliconius hybrid zone: Evidence for phase III of Wright's shifting balance theory? Evolution, 56(10), 1992–1998. 10.1111/j.0014-3820.2002.tb00125.x 12449486

[ece38412-bib-0011] Bolger, A. M. , Lohse, M. , & Usadel, B. (2014). Trimmomatic: A flexible trimmer for Illumina sequence data. Bioinformatics, 30(15), 2114–2120. 10.1093/bioinformatics/btu170 24695404 PMC4103590

[ece38412-bib-0012] Bossert, S. , Gereben‐Krenn, B. A. , Neumayer, J. , Schneller, B. , & Krenn, H. W. (2016). The cryptic Bombus lucorum complex (Hymenoptera: Apidae) in Austria: Phylogeny, distribution, habitat usage and a climatic characterization based on COI sequence data. Zoological Studies, 55, e13. 31966158 10.6620/ZS.2016.55-13 PMC6409445

[ece38412-bib-0013] Bradbury, P. J. , Zhang, Z. , Kroon, D. E. , Casstevens, T. M. , Ramdoss, Y. , & Buckler, E. S. (2007). TASSEL: Software for association mapping of complex traits in diverse samples. Bioinformatics, 23, 26332635. 10.1093/bioinformatics/btm308 17586829

[ece38412-bib-0014] Camacho, C. , Coulouris, G. , Avagyan, V. , Ma, N. , Papadopoulos, J. , Bealer, K. , & Madden, T. L. (2009). BLAST+: Architecture and applications. BMC Bioinformatics, 10(1), 421. 10.1186/1471-2105-10-421 20003500 PMC2803857

[ece38412-bib-0015] Cameron, S. A. , Hines, H. M. , & Williams, P. H. (2007). A comprehensive phylogeny of the bumble bees (*Bombus*). Biological Journal of the Linnean Society, 91(1), 161–188. 10.1111/j.1095-8312.2007.00784.x

[ece38412-bib-0016] Chang, C. C. , Chow, C. C. , Tellier, L. C. , Vattikuti, S. , Purcell, S. M. , & Lee, J. J. (2015). Second‐generation PLINK: Rising to the challenge of larger and richer datasets. Gigascience, 4, 1–16. 10.1186/s13742-015-0047-8 25722852 PMC4342193

[ece38412-bib-0017] Danecek, P. , Auton, A. , Abecasis, G. , Albers, C. A. , Banks, E. , DePristo, M. A. , Handsaker, R. E. , Lunter, G. , Marth, G. T. , Sherry, S. T. , McVean, G. , & Durbin, R. (2011). The variant call format and VCFtools. Bioinformatics, 27(15), 2156–2158. 10.1093/bioinformatics/btr330 21653522 PMC3137218

[ece38412-bib-0018] Darriba, D. , Taboada, G. L. , Doallo, R. , & Posada, D. (2012). jModelTest 2: More models, new heuristics and parallel computing. Nature Methods, 9(8), 772. 10.1038/nmeth.2109 PMC4594756 22847109

[ece38412-bib-0019] Derryberry, E. P. , Derryberry, G. E. , Maley, J. M. , & Brumfield, R. T. (2014). HZAR: Hybrid zone analysis using an R software package. Molecular Ecology Resources, 14(3), 652–663. 24373504 10.1111/1755-0998.12209

[ece38412-bib-0020] Derryberry, G. , & Derryberry, M. G. (2015). Package ‘hzar’.

[ece38412-bib-0021] Duennes, M. A. , Lozier, J. D. , Hines, H. M. , & Cameron, S. A. (2012). Geographical patterns of genetic divergence in the widespread Mesoamerican bumble bee *Bombus ephippiatus* (Hymenoptera: Apidae). Molecular Phylogenetics and Evolution, 64(1), 219–231. 10.1016/j.ympev.2012.03.018 22521295

[ece38412-bib-0022] Egger, B. , Koblmüller, S. , Sturmbauer, C. , & Sefc, K. M. (2007). Nuclear and mitochondrial data reveal different evolutionary processes in the Lake Tanganyika cichlid genus Tropheus. BMC Evolutionary Biology, 7(1), 1–4. 10.1186/1471-2148-7-137 17697335 PMC2000897

[ece38412-bib-0023] Endler, J. A. (1977). Geographic variation, speciation, and clines. Princeton University Press. 409931

[ece38412-bib-0024] Ezray, B. D. , Wham, D. C. , Hill, C. E. , & Hines, H. M. (2019). Unsupervised machine learning reveals mimicry complexes in bumblebees occur along a perceptual continuum. Proceedings of the Royal Society B: Biological Sciences, 286(1910), 20191501. 10.1098/rspb.2019.1501 PMC6742998 31506052

[ece38412-bib-0025] Folmer, O. , Black, M. , Hoeh, W. , Lutz, R. , & Vrijenhoek, R. (1994). DNA primers for amplification of mitochondrial cytochrome c oxidase subunit I from diverse metazoan invertebrates. Molecular Marine Biology and Biotechnology, 3(5), 294–299. 7881515

[ece38412-bib-0026] Franklin, H. J. (1912). The Bombidae of the new world. Transactions of the American Entomological Society, 38(3/4), 177–486.

[ece38412-bib-0027] Frichot, E. , & François, O. (2015). LEA: An R package for landscape and ecological association studies. Methods in Ecology and Evolution, 6(8), 925–929. 10.1111/2041-210X.12382

[ece38412-bib-0028] Funk, D. J. , & Omland, K. E. (2003). Species‐level paraphyly and polyphyly: Frequency, causes, and consequences, with insights from animal mitochondrial DNA. Annual Review of Ecology, Evolution, and Systematics, 34(1), 397–423. 10.1146/annurev.ecolsys.34.011802.132421

[ece38412-bib-0029] Gerth, M. , Geißler, A. N. , & Bleidorn, C. (2011). Wolbachia infections in bees (Anthophila) and possible implications for DNA barcoding. Systematics and Biodiversity, 9(4), 319–327.

[ece38412-bib-0030] Ghisbain, G. , Lozier, J. D. , Rahman, S. R. , Ezray, B. D. , Tian, L. , Ulmer, J. M. , Heraghty, S. D. , Strange, J. P. , Rasmont, P. , & Hines, H. M. (2020). Substantial genetic divergence and lack of recent gene flow support cryptic speciation in a colour polymorphic bumble bee (*Bombus bifarius*) species complex. Systematic Entomology, 45(3), 635–652.

[ece38412-bib-0031] Gompert, Z. , Forister, M. L. , Fordyce, J. A. , & Nice, C. C. (2008). Widespread mito‐nuclear discordance with evidence for introgressive hybridization and selective sweeps in Lycaeides. Molecular Ecology, 17(24), 5231–5244. 19120997 10.1111/j.1365-294X.2008.03988.x

[ece38412-bib-0032] Hijmans, R. J. (2017). geosphere: Spherical Trigonometry. R package version 1.5‐7.

[ece38412-bib-0033] Hines, H. M. (2008). Historical biogeography, divergence times, and diversification patterns of bumble bees (Hymenoptera: Apidae: Bombus). Systematic Biology, 57(1), 58–75. 10.1080/10635150801898912 18275002

[ece38412-bib-0034] Hines, H. M. , & Williams, P. H. (2012). Mimetic colour pattern evolution in the highly polymorphic *Bombus trifasciatus* (Hymenoptera: Apidae) species complex and its comimics. Zoological Journal of the Linnean Society, 166(4), 805–826.

[ece38412-bib-0035] Hudson, R. R. , & Turelli, M. (2003). Stochasticity overrules the “three‐times rule”: Genetic drift, genetic draft, and coalescence times for nuclear loci versus mitochondrial DNA. Evolution, 57(1), 182–190. 12643581 10.1111/j.0014-3820.2003.tb00229.x

[ece38412-bib-0036] Irwin, D. E. (2002). Phylogeographic breaks without geographic barriers to gene flow. Evolution, 56(12), 2383–2394. 10.1111/j.0014-3820.2002.tb00164.x 12583579

[ece38412-bib-0037] Koch, J. B. , Rodriguez, J. , Pitts, J. P. , & Strange, J. P. (2018). Phylogeny and population genetic analyses reveals cryptic speciation in the *Bombus fervidus* species complex (Hymenoptera: Apidae). PLoS One, 13(11), e0207080. 10.1371/journal.pone.0207080 30462683 PMC6248958

[ece38412-bib-0038] Leigh, J. W. , & Bryant, D. (2015). POPART: full‐feature software for haplotype network construction. Methods in Ecology and Evolution, 6(9), 1110–1116.

[ece38412-bib-0039] Li, H. (2011). A statistical framework for SNP calling, mutation discovery, association mapping and population genetical parameter estimation from sequencing data. Bioinformatics, 27(21), 2987–2993. 10.1093/bioinformatics/btr509 21903627 PMC3198575

[ece38412-bib-0040] Li, Z. Y. , Feng, X. , Song, Y. , Shen, Z. R. , & Geng, J. H. (2011). Double infection with Wolbachia strains in three species of bumblebees(Hymenoptera: Apidae). Chinese Journal of Applied Entomology, 48(4), 915–921.

[ece38412-bib-0041] Lozier, J. D. , Strange, J. P. , Stewart, I. J. , & Cameron, S. A. (2011). Patterns of range‐wide genetic variation in six North American bumble bee (Apidae: Bombus) species. Molecular Ecology, 20(23), 4870–4888. 10.1111/j.1365-294X.2011.05314.x 22035452

[ece38412-bib-0042] Mallet, J. (1986). Hybrid zones of Heliconius butterflies in Panama and the stability and movement of warning colour clines. Heredity, 56(2), 191–202. 10.1038/hdy.1986.31

[ece38412-bib-0043] Mallet, J. (2008). Hybridization, ecological races and the nature of species: Empirical evidence for the ease of speciation. Philosophical Transactions of the Royal Society B: Biological Sciences, 363(1506), 2971–2986. 10.1098/rstb.2008.0081 PMC2607318 18579473

[ece38412-bib-0044] Mallet, J. , & Barton, N. H. (1989). Strong natural selection in a warning‐color hybrid zone. Evolution, 43(2), 421–431. 10.1111/j.1558-5646.1989.tb04237.x 28568556

[ece38412-bib-0045] Mallet, J. , Barton, N. , Lamas, G. , Santisteban, J. , Muedas, M. , & Eeley, H. (1990). Estimates of selection and gene flow from measures of cline width and linkage disequilibrium in Heliconius hybrid zones. Genetics, 124(4), 921–936. 10.1093/genetics/124.4.921 2323556 PMC1203983

[ece38412-bib-0046] Mallet, J. , McMillan, W. O. , & Jiggins, C. D. (1998). Mimicry and warning color at the boundary between races and species. In D. J. Howard , & S. H. Berlocher (Eds.), Endless forms: Species and speciation (pp. 390–403). Oxford University Press.

[ece38412-bib-0047] Martinet, B. , Lecocq, T. , Brasero, N. , Biella, P. , Urbanova, K. , Valterova, I. , Cornalba, M. , Gjershaug, J. O. , Michez, D. , & Rasmont, P. (2018). Following the cold: geographical differentiation between interglacial refugia and speciation in the arcto‐alpine species complex *Bombus monticola* (Hymenoptera: Apidae). Systematic Entomology, 43(1), 200–217.

[ece38412-bib-0048] Martinet, B. , Lecocq, T. , Brasero, N. , Gerard, M. , Urbanová, K. , Valterová, I. , Gjershaug, J. O. , Michez, D. , & Rasmont, P. (2019). Integrative taxonomy of an arctic bumblebee species complex highlights a new cryptic species (Apidae: Bombus). Zoological Journal of the Linnean Society, 187(3), 599–621. 10.1093/zoolinnean/zlz041

[ece38412-bib-0049] Masui, S. , Sasaki, T. , & Ishikawa, H. (1997). groE‐homologous operon of Wolbachia, an intracellular symbiont of arthropods: A new approach for their phylogeny. Zoological Science, 14(4), 701–706. 9401467 10.2108/zsj.14.701

[ece38412-bib-0050] Matsuura, E. T. , Niki, Y. , & Chigusa, S. I. (1993). Temperature‐dependent selection in the transmission of mitochondrial DNA in *Drosophila* . The Japanese Journal of Genetics, 68(2), 127–135. 10.1266/jjg.68.127 8369137

[ece38412-bib-0051] McKenna, A. , Hanna, M. , Banks, E. , Sivachenko, A. , Cibulskis, K. , Kernytsky, A. , Garimella, K. , Altshuler, D. , Gabriel, S. , Daly, M. , & DePristo, M. A. (2010). The Genome Analysis Toolkit: A MapReduce framework for analyzing next‐generation DNA sequencing data. Genome Research, 20(9), 1297–1303. 10.1101/gr.107524.110 20644199 PMC2928508

[ece38412-bib-0052] Miller, M. P. , Bellinger, M. R. , Forsman, E. D. , & Haig, S. M. (2006). Effects of historical climate change, habitat connectivity, and vicariance on genetic structure and diversity across the range of the red tree vole (*Phenacomys longicaudus*) in the Pacific Northwestern United States. Molecular Ecology, 15(1), 145–159. 10.1111/j.1365-294X.2005.02765.x 16367837

[ece38412-bib-0053] Miller, M. A. , Pfeiffer, W. , & Schwartz, T. (2010). Creating the CIPRES Science Gateway for inference of large phylogenetic trees. In 2010 gateway computing environments workshop (GCE) (pp. 1–8).

[ece38412-bib-0054] Milliron, H. E. (1971). A monograph of the Western Hemisphere bumblebees (Hymenoptera: Apidae; Bombinae). I. The. Memoirs of the Entomological Society of Canada, 103(S82), 1–80. 10.4039/entm10382fv

[ece38412-bib-0055] Owen, R. E. (1986). Gene frequency clines at X‐linked or haplodiploid loci. Heredity, 57(2), 209–219. 10.1038/hdy.1986.111 3781871

[ece38412-bib-0056] Owen, R. E. , & Plowright, R. C. (1980). Abdominal pile color dimorphism in the bumble bee, *Bombus melanopygus* . Journal of Heredity, 71(4), 241–247. 10.1093/oxfordjournals.jhered.a109357

[ece38412-bib-0057] Owen, R. E. , Whidden, T. L. , & Plowright, R. C. (2010). Genetic and morphometric evidence for the conspecific status of the bumble bees, *Bombus melanopygus* and *Bombus edwardsii* . Journal of Insect Science, 10(1), 109. 20874396 10.1673/031.010.10901 PMC3016928

[ece38412-bib-0058] Pascar, J. , & Chandler, C. H. (2018). A bioinformatics approach to identifying Wolbachia infections in arthropods. PeerJ, 6, e5486. 30202647 10.7717/peerj.5486 PMC6126470

[ece38412-bib-0059] Pavlova, A. , Amos, J. N. , Joseph, L. , Loynes, K. , Austin, J. J. , Keogh, J. S. , Stone, G. N. , Nicholls, J. A. , & Sunnucks, P. (2013). Perched at the mito‐nuclear crossroads: Divergent mitochondrial lineages correlate with environment in the face of ongoing nuclear gene flow in an Australian bird. Evolution, 67(12), 3412–3428. 10.1111/evo.12107 24299397

[ece38412-bib-0061] Posada, D. , & Crandall, K. A. (2001). Intraspecific gene genealogies: Trees grafting into networks. Trends in Ecology & Evolution, 16(1), 37–45. 10.1016/S0169-5347(00)02026-7 11146143

[ece38412-bib-0062] Quek, S. P. , Davies, S. J. , Itino, T. , & Pierce, N. E. (2004). Codiversification in an ant‐plant mutualism: Stem texture and the evolution of host use in Crematogaster (Formicidae: Myrmicinae) inhabitants of Macaranga (Euphorbiaceae). Evolution, 58(3), 554–570. 10.1111/j.0014-3820.2004.tb01678.x 15119439

[ece38412-bib-0063] R Core Team . (2017). R: A language and environment for statistical computing. R Foundation for Statistical Computing. Retrieved from https://www.R‐project.org

[ece38412-bib-0064] Rahman, S. R. , Terranova, T. , Tian, L. , & Hines, H. M. (2021). Developmental transcriptomics reveals a gene network driving mimetic color variation in a bumble bee. Genome Biology and Evolution, 13(6), evab080. 10.1093/gbe/evab080 PMC8220310 33881508

[ece38412-bib-0065] Remington, C. L. (1968). Suture‐zones of hybrid interaction between recently joined biotas. In T. Dobzhansky , M. K. Hecht , & W. C. Steere (Eds.), Evolutionary biology (pp. 321–428). Springer.

[ece38412-bib-0067] Roca, A. L. , Georgiadis, N. , & O'Brien, S. J. (2005). Cytonuclear genomic dissociation in African elephant species. Nature Genetics, 37(1), 96–100. 10.1038/ng1485 15592471

[ece38412-bib-0068] Ronquist, F. , Teslenko, M. , Van Der Mark, P. , Ayres, D. L. , Darling, A. , Höhna, S. , Larget, B. , Liu, L. , Suchard, M. A. , & Huelsenbeck, J. P. (2012). MrBayes 3.2: Efficient Bayesian phylogenetic inference and model choice across a large model space. Systematic Biology, 61(3), 539–542. 10.1093/sysbio/sys029 22357727 PMC3329765

[ece38412-bib-0069] Rosser, N. , Dasmahapatra, K. K. , & Mallet, J. (2014). Stable Heliconius butterfly hybrid zones are correlated with a local rainfall peak at the edge of the Amazon basin. Evolution, 68(12), 3470–3484. 25311415 10.1111/evo.12539

[ece38412-bib-0070] Soltis, D. E. , Gitzendanner, M. A. , Strenge, D. D. , & Soltis, P. S. (1997). Chloroplast DNA intraspecific phylogeography of plants from the Pacific Northwest of North America. Plant Systematics and Evolution, 206(1–4), 353–373. 10.1007/BF00987957

[ece38412-bib-0071] Swenson, N. G. , & Howard, D. J. (2005). Clustering of contact zones, hybrid zones, and phylogeographic breaks in North America. The American Naturalist, 166(5), 581–591. 10.1086/491688 16224723

[ece38412-bib-0072] Thorp, R. W. , Horning, D. S. , & Dunning, L. L. (1983). Bumble bees and cuckoo bumble bees of California (Hymenoptera, Apidae). University of California Press.

[ece38412-bib-0073] Thurman, T. J. , Szejner‐Sigal, A. , & McMillan, W. O. (2019). Movement of a Heliconius hybrid zone over 30 years: A Bayesian approach. Journal of Evolutionary Biology, 32(9), 974–983. 31216075 10.1111/jeb.13499

[ece38412-bib-0074] Tian, L. , Rahman, S. R. , Ezray, B. D. , Franzini, L. , Strange, J. P. , Lhomme, P. , & Hines, H. M. (2019). A homeotic shift late in development drives mimetic color variation in a bumble bee. Proceedings of the National Academy of Sciences of the United States of America, 116(24), 11857–11865. 10.1073/pnas.1900365116 31043564 PMC6575597

[ece38412-bib-0075] Toews, D. P. , & Brelsford, A. (2012). The biogeography of mitochondrial and nuclear discordance in animals. Molecular Ecology, 21(16), 3907–3930. 10.1111/j.1365-294X.2012.05664.x 22738314

[ece38412-bib-0076] Twomey, E. , Vestergaard, J. S. , Venegas, P. J. , & Summers, K. (2016). Mimetic divergence and the speciation continuum in the mimic poison frog *Ranitomeya imitator* . The American Naturalist, 187(2), 205–224. 10.1086/684439 26807748

[ece38412-bib-0077] Wenseleers, T. , Ito, F. , Van Borm, S. , Huybrechts, R. , Volckaert, F. , & Billen, J. (1998). Widespread occurrence of the microorganism Wolbachia in ants. Proceedings of the Royal Society of London. Series B: Biological Sciences, 265(1404), 1447–1452. 10.1098/rspb.1998.0456 PMC1689219 9721689

[ece38412-bib-0078] Wickham, H. (2016). ggplot2: Elegant graphics for data analysis. Springer.

[ece38412-bib-0079] Williams, P. (2007). The distribution of bumblebee colour patterns worldwide: Possible significance for thermoregulation, crypsis, and warning mimicry. Biological Journal of the Linnean Society, 92(1), 97–118. 10.1111/j.1095-8312.2007.00878.x

[ece38412-bib-0080] Williams, P. H. , Altanchimeg, D. , Byvaltsev, A. , De Jonghe, R. , Jaffar, S. , Japoshvili, G. , Kahono, S. , Liang, H. , Mei, M. , Monfared, A. , Nidup, T. , Raina, R. , Ren, Z. , Thanoosing, C. , Zhao, Y. , & Orr, M. C. (2020). Widespread polytypic species or complexes of local species? Revising bumblebees of the subgenus Melanobombus world‐wide (Hymenoptera, Apidae, Bombus). European Journal of Taxonomy, 719, 1–20. 10.5852/ejt.2020.719.1107

[ece38412-bib-0081] Yang, D. S. , & Kenagy, G. J. (2009). Nuclear and mitochondrial DNA reveal contrasting evolutionary processes in populations of deer mice (*Peromyscus maniculatus*). Molecular Ecology, 18(24), 5115–5125. 10.1111/j.1365-294X.2009.04399.x 19912541

[ece38412-bib-0082] Zha, X. , Zhang, W. , Zhou, C. , Zhang, L. , Xiang, Z. , & Xia, Q. (2014). Detection and characterization of Wolbachia infection in silkworm. Genetics and Molecular Biology, 37(3), 573–580. 10.1590/S1415-47572014000400014 25249781 PMC4171764

[ece38412-bib-0083] Zink, R. M. , & Barrowclough, G. F. (2008). Mitochondrial DNA under siege in avian phylogeography. Molecular Ecology, 17(9), 2107–2121. 10.1111/j.1365-294X.2008.03737.x 18397219

