# Supplementary material for: Mito‐nuclear discordance at a mimicry color transition zone in bumble bee Bombus melanopygus
Source: Ecol Evol. 2021 Dec 8;11(24):18151–68. doi: 10.1002/ece3.8412 (PMC8717287; doi:10.1002/ece3.8412)
Supplement: Supplementary file 1 — Figure S1 [file ECE3-11-18151-s001.docx]

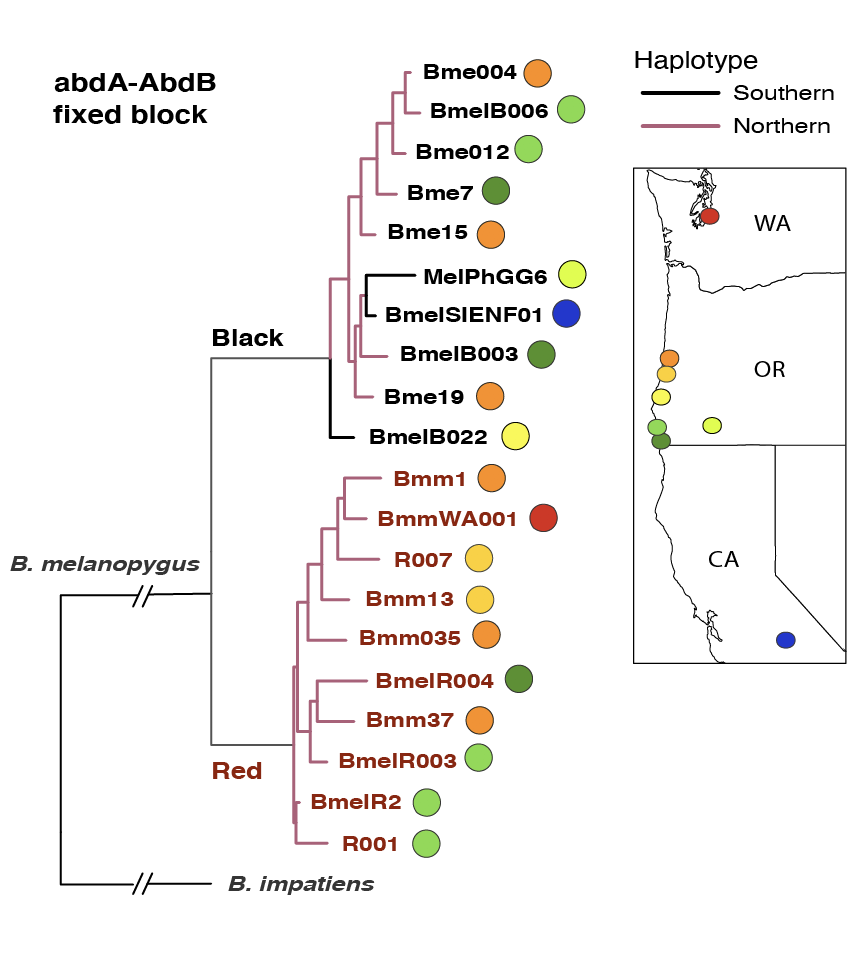


**Figure S1**. NJ phylogeny in the ~18Kb locus that drives coloration, including all

SNPs with no more than 20% missing data. Branch color represents haplotype, name color represents color phenotype, and circles represent geographic location as outlined on the map.
