# Supplementary material for: Mito‐nuclear discordance at a mimicry color transition zone in bumble bee Bombus melanopygus
Source: Ecol Evol. 2021 Dec 8;11(24):18151–68. doi: 10.1002/ece3.8412 (PMC8717287; doi:10.1002/ece3.8412)
Supplement: Supplementary file 2 — Figure S2 [file ECE3-11-18151-s004.docx]

**
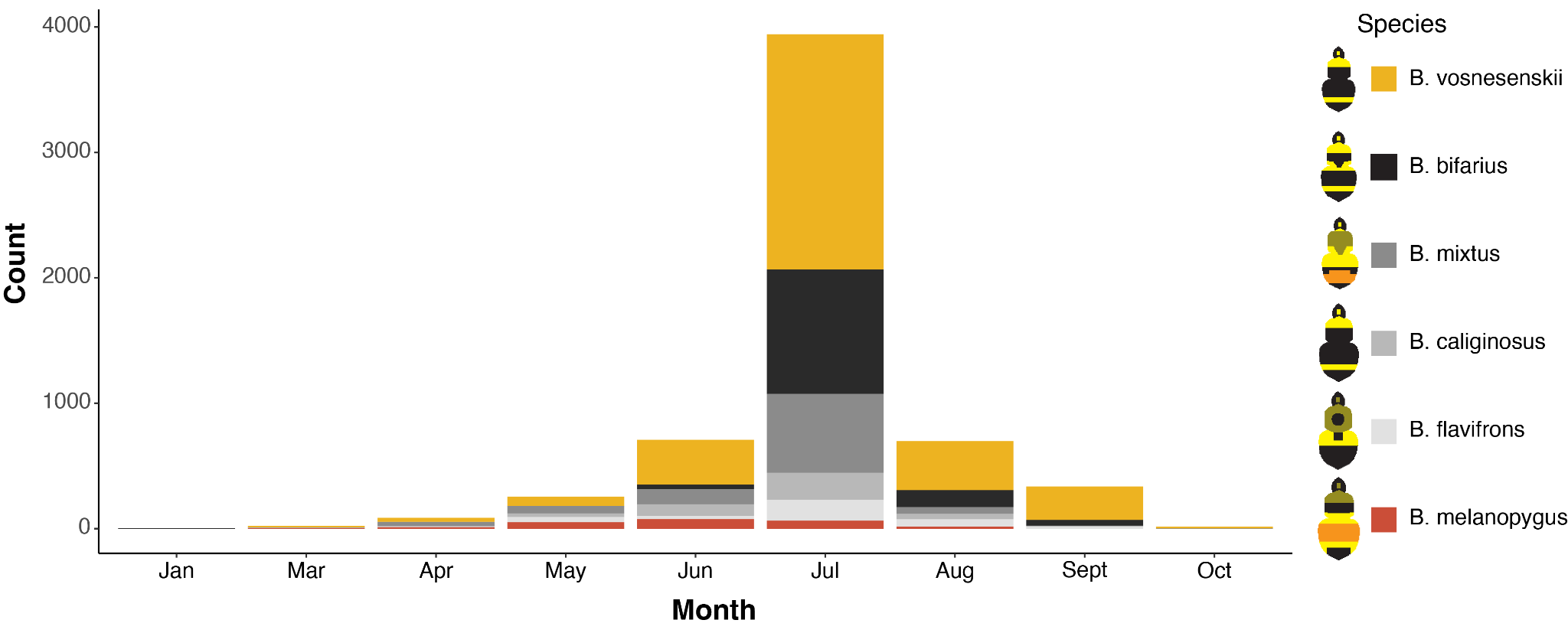
**

**Figure S2:** Abundance of common bumble bee species records by month within the *B. melanopygus* hybrid zone region. Preserved specimen records were extracted from the Global Biodiversity Information Facility (GBIF) for the most common bumble bee species in this region, *B. vosnesenskii* (GBIF.org (21 October 2021) GBIF Occurrence Download https://doi.org/10.15468/dl.zrzmnd), *B. bifarius* (GBIF.org (21 October 2021) GBIF Occurrence Download https://doi.org/10.15468/dl.pwsvjr), *B. mixtus* (GBIF.org (21 October 2021) GBIF Occurrence Download https://doi.org/10.15468/dl.jvjks6), *B. caliginosus* (GBIF.org (21 October 2021) GBIF Occurrence Download https://doi.org/10.15468/dl.y8tgtk), *B. flavifron*s (GBIF.org (21 October 2021) GBIF Occurrence Download https://doi.org/10.15468/dl.6h9t8r), as well as *B. melanopygus* (GBIF.org (21 October 2021) GBIF Occurrence Download https://doi.org/10.15468/dl.5pbz2y) to understand which species and color forms are numerically dominant within the *B. melanopygus* hybrid zone region. Analysis was limited to records contained within 41 and 45 degrees North and -122 and -125 degrees West. February was excluded from the analysis as records were not present.
