# Supplementary material for: Mito‐nuclear discordance at a mimicry color transition zone in bumble bee Bombus melanopygus
Source: Ecol Evol. 2021 Dec 8;11(24):18151–68. doi: 10.1002/ece3.8412 (PMC8717287; doi:10.1002/ece3.8412)
Supplement: Supplementary file 3 — Table S1 [file ECE3-11-18151-s003.pdf]

Table S1. Information for specimens used in this study including collection information, abdominal color variants, color locus alleles (All.), COI group assignment including affinity to northern and southern clades and individual sequence haplotypes in the haplotype network (COI Net.), and Genbank numbers for specimens used for COI mitochondrial (Mt. Seq), color locus, and genome sequencing.

ID	Species	Type	Sex	Color	All.	Latitude	Longitude	Locality	Coll. Date	COI Haplo.	COI Net.	Mt. Seq	Color	Genome (SRA)
109 B	melanopygus	Reared	M	Black	b	42.4097	-124.4202	OR: Chase bank, Gold Beach	2/3/18	Northern	H9	OL435630		
109 R	melanopygus	Reared	M	Red	r	42.4097	-124.4202	OR: Chase bank, Gold Beach	2/3/18	Northern	H9	OL435620		
122_2 B	melanopygus	Reared	M	Black	b	43.9751	-124.1195	OR: Peace Harbor Medical Center, Florence	2/4/18	Northern	H9	OL435636		
122_2 R	melanopygus	Reared	M	Red	r	43.9751	-124.1195	OR: Peace Harbor Medical Center, Florence	2/4/18	Northern	H9	OL435619		
126 B	melanopygus	Reared	M	Black	b	43.9751	-124.1195	OR: Peace Harbor Medical Center, Florence	2/4/18	Northern	H9	OL435632		
126 R	melanopygus	Reared	M	Red	r	43.9751	-124.1195	OR: Peace Harbor Medical Center, Florence	2/4/18	Northern	H9	OL435637		
136 R	melanopygus	Reared	Q	Red		43.9751	-124.1195	OR: Peace Harbor Medical Center, Florence	2/4/18	Northern	H9	OL435638		
146 B	melanopygus	Reared	M	Black	b	42.45155	-123.3325	OR: Savage Street/Highland Ave, Grants Pass	2/6/18	Northern	H9	OL435641		
146 R	melanopygus	Reared	M	Red	r	42.45155	-123.3325	OR: Savage Street/Highland Ave, Grants Pass	2/6/18	Northern	H9	OL435628		
2018_92_B_Q	melanopygus	Wild	Q	Black		42.404	-124.423	OR: Gold Beach Inn	2/2/17	Southern	H3	OL435676		
2018_92RWACG	melanopygus	Reared	W	Red		42.404	-124.423	OR: Gold Beach Inn		Southern	H3	OL435661		
2018_93B_CG	melanopygus	Reared	W	Black		42.4097	-124.4202	OR: Chase bank, Gold Beach	2/2/17	Northern	H9	OL435691		
35R2019	melanopygus	Wild	Q	Red		42.4097	-124.4202	OR: Chase bank, Gold Beach	1/29/19	Northern	H9	OL435681		
58B	melanopygus	Reared	Q	Black		41.074733	-124.141583	CA: Redwood Hwy, 65mi S of Crescent City	2/1/17	Southern	H3	OL435667		
82B2019	melanopygus	Wild	Q	Black		43.9751	-124.1195	OR: Peace Harbor Medical Center, Florence	1/30/19	Northern	H9	OL435680		
85R2019	melanopygus	Wild	Q	Red		41.753735	-124.192754	CA: Kin Khao Thai resturant, Crescent City	1/31/19	Northern	H9	OL435569		
Bme004	melanopygus	Wild	M	Black	b	43.9561167	-124.1374444	OR: Florence, Dunes National Rec. Area	6/1/14	Northern	H9	OL435593	OL461844	SRR8700086
Bme007	melanopygus	Wild	M	Black	b	42.1043972	-124.34695	OR: Cape Ferello	6/3/14	Northern	H9	OL435686	OL461848	SRR8700082
Bme012	melanopygus	Wild	M	Black	b	42.3288278	-124.4265417	OR: Cape Sebastian	6/4/14	Northern	H9	OL435618	OL461845	SRR8700085
Bme015	melanopygus	Wild	M	Black	b	43.9563833	-124.1371056	OR: Florence, Dunes National Rec. Area	6/9/14	Northern	H9	OL435609	OL461846	SRR8700084
Bme019	melanopygus	Wild	M	Black	b	44.0518139	-124.1095361	OR: Florence, Sutton Recreational Area	6/10/14	Northern	H9	OL435608	OL461847	SRR8700083
BmelB001	melanopygus	Wild	M	Black	b	42.1065111	-124.3466306	OR: Cape Ferello	6/18/13	Northern	H9	OL435695	OL461850	
BmelB004	melanopygus	Wild	M	Black	b	42.3289	-124.4258611	OR: Cape Sebastian	6/18/13	Northern	H9	OL435594	OL461852	
BmelB005	melanopygus	Wild	M	Black	b	42.3289	-124.4258611	OR: Cape Sebastian	6/18/13	Northern	H9	OL435601	OL461888	
BmelB006	melanopygus	Wild	M	Black	b	42.3289	-124.4258611	OR: Cape Sebastian	6/19/13	Northern	H9	OL435600	OL461851	SRR8700079
BmelB002	melanopygus	Wild	M	Black	b	42.1065111	-124.3466306	OR: Cape Ferello	6/18/13	Southern	H3	OL435678		
BmelB003	melanopygus	Wild	M	Black	b	42.1065111	-124.3466306	OR: Cape Ferello	6/18/13	Northern	H7	OL435591	OL461849	SRR8700080
BmelB021	melanopygus	Wild	M	Black	b	42	-122.77	CA/OR: Beaver Creek Rd, Hwy 96-Mt. Ashland	7/15/15	Northern	H9	OL435573	OL461886	
BmelB022	melanopygus	Wild	M	Black	b	43.209836	-124.31046	OR: 16001-16015 W. Beaver Hill Rd, Coos Bay	6/7/15	Southern	H4	OL435679	OL461887	SRR16832690
BmelB023	melanopygus	Wild	M	Red		43.956383	-124.1371056	OR: Florence, Dunes National Rec.Area	6/8/14				OL461896	
BmelB026	melanopygus	Wild	M	Black	b	43.209836	-124.31046	OR: 16001-16015 W. Beaver Hill Rd, Coos Bay	6/7/15	Northern	H9	OL435653	OL461863	
BmelB027	melanopygus	Wild	F	Black		42.0735861	-122.6048889	OR: Ashland, Mount Ashland	6/7/14	Southern	H3	OL435582	OL461864	
BmelB028	melanopygus	Wild	M	Black		44.0518139	-124.1095361	OR: Florence, Sutton Recreational Area	6/10/14				OL461865	
BmelB029	melanopygus	Wild	M	Black	b	43.9563833	-124.1371056	OR: Florence, Dunes National Rec. Area	6/9/14	Northern	H6	OL435599	OL461866	
BmelB030	melanopygus	Wild	M	Black		42.1063056	-124.3464917	OR: Cape Ferrelo	6/3/14				OL461867	

BmelB031	melanopygus	Wild	M	Black		43.9563833	-124.1371056	OR: Florence, Dunes National Rec.Area	6/1/14				OL461868	
BmelB032	melanopygus	Wild	M	Black		44.0518139	-124.1095361	OR: Florence, Sutton Recreational Area	6/10/14				OL461869	
BmelB036	melanopygus	Wild	M	Black		42.3289	-124.4258611	OR: Cape Sebastian	6/19/13				OL461870	
BmelB037	melanopygus	Wild	M	Black		42.1065111	-124.3466306	OR: Cape Ferrelo	6/18/13				OL461871	
BmelB038	melanopygus	Wild	Q	Black	bb	40.7565944	-124.1646333	CA: Eureka	3/2/15	Southern	H3	OL435583	OL461872	
BmelB051	melanopygus	Wild	M	Black	b	41.797607	-124.081845	CA: nr. Jedediah Smith State Park	6/6/15	Northern	H6	OL435633		
BmelB057	melanopygus	Wild	M	Black	b	42.1065111	-124.3466306	OR: Cape Ferello	6/19/13	Northern	H9	OL435571		
BmelB061	melanopygus	Wild	M	Black	b	44.0518139	-124.1095361	OR: Florence, Sutton Recreational Area	6/10/14	Northern	H9	OL435693		
BmelB062	melanopygus	Wild	M	Black	b	41.34678	-122.24143	CA: Mt. Shasta, Everitt Memorial Hwy	7/15/15	Southern	H3	OL435668		
BmelB063	melanopygus	Wild	M	Black	b	40.3273	-123.925468	CA: Marin Garden Club Group Camp	6/5/15	Southern	H3	OL435584		
BmelB064	melanopygus	Wild	F	Black	bb	41.769369	-124.155144	CA: Aubell Ln.- Elk Valley Rd, Crescent City	6/6/15	Southern	H4	OL435671	OL461873	
BmelB066	melanopygus	Wild	M	Black	b	42.1065111	-124.3466306	OR: Cape Ferello	6/18/13	Northern	H9	OL435617	OL461876	
BmelB067	melanopygus	Wild	M	Black		42.1065111	-124.3466306	OR: Cape Ferrelo	6/18/13				OL461877	
BmelB068	melanopygus	Wild	M	Black		*40.3273*	-123.9254680	CA: Marin Garden Club Group Camp	6/5/15				OL461878	
BmelB069	melanopygus	Wild	M	Black		43.9563833	-124.1371056	OR: Florence, Dunes National Rec. Area	6/1/14				OL461879	
BmelB070	melanopygus	Wild	M	Black	b	42.1045972	-124.3467667	OR: Cape Ferello	6/3/14	Northern	H9	OL435580	OL461880	
BmelB072	melanopygus	Wild	M	Black		43.9563833	-124.1371056	OR: Florence, Dunes National Rec. Area	6/1/14				OL461881	
BmelB073	melanopygus	Wild	M	Black		42.1065111	-124.3466306	OR: Cape Ferrelo	6/18/13				OL461882	
BmelB074	melanopygus	Wild	M	Black		41.34678	-122.2414300	CA: Mt. Shasta, Everitt Memorial Hwy,	7/15/15				OL461883	
BmelB100	melanopygus	Wild	M	Black	b	42.081689	-122.706948	OR: Mt. Ashland Bull Gap Trailhead	5/28/16	Northern	H9	OL435683	OL461884	
BmelB101	melanopygus	Wild	M	Black	b	43.029071	-124.076219	OR: Hwy 42 W (41 ish mi to 101 N)	5/29/16	Northern	H9	OL435634		
BmelB102	melanopygus	Wild	M	Black	b	42.687131	-124.437234	OR: Humbug Mt. Day use area	5/25/16	Northern	H9	OL435574	OL461885	
BmelB103	melanopygus	Wild	M	Black	b	41.855586	-124.072924	CA: Jedediah Redwoods State Park	5/27/16	Northern	H9	OL435652		
BmelB202	melanopygus	Wild	F	Black	bb	37.3972864	-119.5954027	CA: Sierra National Forest	6/10/17	Southern	H3	OL435656	OL461875	
BmelB203	melanopygus	Wild	M	Black	b	36.1363782	-118.5407347	CA: Sequoia National Forest	6/24/17	Southern	H3	OL435657		
BmelBOTM045	melanopygus	Wild	M	Black	b	41.1782051	-124.07747	CA: Patrick's Point	5/26/16	Southern	H3	OL435670	OL461891	
BmelBOTM047	melanopygus	Wild	M	Black	b	41.2215212	-124.0931031	CA: .nr.Humboldt Lagoon St.Pk.	5/26/16	Southern	H4	OL435669	OL461892	
BmelBOTM049	melanopygus	Wild	M	Black	b	42.100447	-124.3470295	OR: Lone Ranch Picnic Area nr. Cape Ferello	5/27/16	Northern	H9	OL435603	OL461893	
BmelBOTM050	melanopygus	Wild	M	Black	b	42.687131	-124.437234	OR: Humbug Mt. Day use area	5/25/16	Northern	H9	OL435602	OL461874	
BmelBOTM051	melanopygus	Wild	M	Black	b	42.2847724	-124.4070977	OR: Pistol River Loop, N. Bank Pistol River	5/25/16	Northern	H9	OL435605	OL461894	
BmelR001	melanopygus	Wild	M	Red	r	42.4648389	-124.3487806	OR: Gold Beach, Rogue River	6/19/13	Northern	H9	OL435692	OL461853	SRR8700096
BmelR002	melanopygus	Wild	M	Red	r	42.3289	-124.4258611	OR: Cape Sebastian	6/18/13	Northern	H9	OL435587	OL461862	SRR8700094
BmelR003	melanopygus	Wild	M	Red	r	42.3289	-124.4258611	OR: Cape Sebastian	6/18/13	Northern	H9	OL435589	OL461860	SRR8700092
BmelR004	melanopygus	Wild	M	Red	r	42.1065111	-124.3466306	OR: Cape Ferello	6/18/13	Northern	H9	OL435586	OL461861	SRR8700091
BmelR005	melanopygus	Wild	M	Red	r	42.1065111	-124.3466306	OR: Cape Ferello	6/18/13	Northern	H9	OL435644		SRR8700093
BmelR006	melanopygus	Wild	M	Red	r	43.6741	-124.1676194	OR: Reedsport, Umpqua River Lighthouse	6/15/13	Northern	H9	OL435625	OL461904	
BmelR007	melanopygus	Wild	M	Red	r	43.8121667	-124.1589972	OR: Reedsport, Tahkenitch Creek Trailhead Rd.	6/15/13	Northern	H9	OL435604	OL461854	SRR8700095
BmelR010	melanopygus	Wild	M	Red	r	45.30289	-121.79368	OR: Mirror Lake Trail at Mt. Hood	7/18/15	Northern	H9	OL435612		
BmelR011	melanopygus	Wild	M	Red		44.79286	-121.83786	OR: NF-4420 Rd. to Olalie Lake	7/17/15				OL461898	
BmelR012	melanopygus	Wild	M	Red		43.209836	-124.31046	OR: 16001-16015 W. Beaver Hill Rd, Coos Bay	6/7/15				OL461900	
BmelR013	melanopygus	Wild	M	Red	r	42.3289	-124.4258611	OR: Cape Sebastian	6/19/13	Northern	H9	OL435690	OL461901	
BmelR014	melanopygus	Wild	F	Red	rb	42.0735861	-122.6048889	OR: Ashland, Mount Ashland	6/7/14	Northern	H9	OL435577		
BmelR016	melanopygus	Wild	M	Red		42.328608	-124.4280056	OR: Cape Sebastian	6/4/14				OL461906	
BmelR017	melanopygus	Wild	M	Red		43.658211	-124.1886111	OR: Reedsport, Umpqua River Lighthouse	6/19/13				OL461907	
BmelR018	melanopygus	Wild	F	Red	rb	42.1043306	-124.3465667	OR: Cape Ferello	6/3/14	Northern	H9	OL435613		
BmelR040	melanopygus	Wild	F	Red	rb	44.504979	-123.551079	OR: Mary's Peak	6/10/15	Northern	H9	OL435623	OL461908	

BmelR041	melanopygus	Wild	W	Red		43.797869	-124.147915	OR: Takenitch Lake	6/7/15				OL461909	
BmelR042	melanopygus	Wild	M	Red	r	42.3289	-124.4258611	OR: Cape Sebastian	6/19/13	Northern	H9	OL435616	OL461910	
BmelR043	melanopygus	Wild	M	Red		42.106511	-124.3466306	OR: Cape Ferello	6/17/13				OL461911	
BmelR044	melanopygus	Wild	M	Red	r	42.1043306	-124.3465667	OR: Cape Ferello	6/3/14	Northern	H9	OL435622	OL461912	
BmelR045	melanopygus	Wild	M	Red		42.328803	-124.4284667	OR: Cape Sebastian	6/4/14				OL461913	
BmelR084	melanopygus	Wild	M	Red	r	42.1065556	-124.3464944	OR: Cape Ferello	6/3/14	Northern	H9	OL435649		
BmelR091	melanopygus	Wild	M	Red	r	42.1065111	-124.3466306	OR: Cape Ferello	6/17/13	Northern	H9	OL435639		
BmelR093	melanopygus	Wild	F	Red	rr	43.209836	-124.31046	OR: 16001-16015 W. Beaver Hill Rd, Coos Bay	6/7/15	Northern	H9	OL435627	OL461914	
BmelR094	melanopygus	Wild	F	Red	rb	44.504979	-123.551079	OR: Mary's Peak	6/10/15	Northern	H9	OL435631		
BmelR096	melanopygus	Wild	F	Red	rb	44.504979	-123.551079	OR: Mary's Peak	6/10/15	Northern	H9	OL435645		
BmelR097	melanopygus	Wild	F	Red	rb	44.504979	-123.551079	OR: Mary's Peak	6/10/15	Northern	H9	OL435626		
BmelR098	melanopygus	Wild	F	Red	rr	47.322326	-122.397293	WA: Palisades Park, Federal Way	6/12/15	Northern	H9	OL435682	OL461915	
BmelR104	melanopygus	Wild	Q	Red		43.955644	-124.1387944	OR: Florence, Dunes National Rec. Area	3/4/14				OL461917	
BmelR105	melanopygus	Wild	M	Red	r	42.1065111	-124.3466306	OR: Cape Ferello	6/17/13	Northern	H5	OL435643		
BmelR109	melanopygus	Wild	M	Red		43.658211	-124.1886111	OR: Reedsport, Umpqua River Lighthouse	6/19/13				OL461918	
BmelR110	melanopygus	Wild	M	Red	r	42.1065111	-124.3466306	OR: Cape Ferello	6/17/13	Northern	H9	OL435642	OL461919	
BmelR111	melanopygus	Wild	M	Red		43.6741	-124.1676194	OR: Reedsport, Umpqua River Lighthouse	6/15/13				OL461920	
BmelR112	melanopygus	Wild	M	Red		43.6582111	-124.1886111	OR: Reedsport, Umpqua River Lighthouse	6/15/13				OL461921	
BmelR113	melanopygus	Wild	M	Red		43.812164	-124.1589970	OR: Tahkenitch Creek Trailhead Rd.	6/16/13				OL461922	
BmelR114	melanopygus	Wild	W	Red		44.504979	-123.5510790	OR: Mary's Peak	6/10/15				OL461923	
BmelR115	melanopygus	Wild	Q	Red		43.9556444	-124.1387944	OR: Florence, Dunes National Rec. Area	3/4/14				OL461924	
BmelR116	melanopygus	Wild	M	Red		44.0518139	-124.1095361	OR: Florence, Sutton Recreational Area	6/10/14				OL461925	
BmelR117	melanopygus	Wild	Q	Red	rb	43.956923	-124.141151	OR: Florence	2/19/15	Northern	H9	OL435596		
BmelR118	melanopygus	Wild	M	Red		43.9561167	-124.1374444	OR: Florence, Dunes National Rec.Area	6/9/14				OL461926	
BmelR119	melanopygus	Wild	M	Red		43.658211	-124.1886111	OR: Reedsport, Umpqua River Lighthouse	6/19/13				OL461897	
BmelR121	melanopygus	Wild	Q	Red		43.956923	-124.141151	OR: Florence	2/19/15				OL461899	
BmelR140	melanopygus	Wild	M	Red	r	42.081689	-122.706948	OR: Mt. Ashland area, 5219-5311 Old Hwy 99S	5/28/16	Northern	H9	OL435684	OL461902	
BmelR141	melanopygus	Wild	M	Red	r	43.029071	-124.076219	OR: Hwy 42 W (41 ish mi to 101 N)	5/29/16	Northern	H7	OL435648	OL461903	
BmelR142	melanopygus	Wild	M	Red	r	42.687131	-124.437234	OR: Humbug Mt. Day use area	5/25/16	Northern	H9	OL435646		
BmelR143	melanopygus	Wild	M	Red	r	41.855586	-124.072924	CA: Jedediah Redwoods State Park	5/27/16	Northern	H9	OL435651		
BmelR200	melanopygus	Wild	M	Red	r	43.01821	-109.75566	WY: Wind river range, Elkhart Park	8/10/16	Northern	H9	OL435611	OL461927	
BmelR201	melanopygus	Wild	M	Red	r	40.84828	-109.645073	UT: Ashley National Forest, Moose Pond		Northern	H9	OL435621	OL461928	
BmelRCF	melanopygus	Wild	M	Red	r	42.1065111	-124.3466306	OR: Cape Ferello	5/25/16	Northern	-	OL435570	OL461929	
BmelRInt03	melanopygus	Wild	F	Red	rb	42.687131	-124.437234	OR: Humbug Mt. Day use area	5/25/16	Northern	H9	OL435624		
BmelRint04	melanopygus	Wild	M	Red	r	41.855586	-124.072924	CA: Jedediah Redwoods State Park	5/27/16	Northern	H9	OL435629	OL461905	
BmelRint1	melanopygus	Wild	F	Red	rb	42.081689	-122.706948	OR: Mt. Ashland area, 5219-5311 Old Hwy 99S	5/28/16	Northern	H9	OL435610		
BmelRint2	melanopygus	Wild	Q	Red	rb	43.029071	-124.076219	OR: Hwy 42 W (41 ish mi to 101 N)	6/7/16	Northern	H9	OL435647		
BmelROTM042	melanopygus	Wild	M	Red	r	42.0649176	-124.3026239	OR: Rest Area N of Brookings	5/27/16	Northern	H9	OL435595	OL461916	
Bmm_WA001	melanopygus	Wild	M	Red	r	47.038125	-122.901424	WA: Olympia	2014	Northern	H9	OL435614	OL461859	SRR8700097
Bmm001	melanopygus	Wild	M	Red	r	43.9561167	-124.1374444	OR: Florence, Dunes National Rec. Area	6/1/14	Northern	H9	OL435689	OL461857	SRR8700090
Bmm002	melanopygus	Wild	M	Red	r	43.9561167	-124.1374444	OR: Florence, Dunes National Rec. Area	6/1/14	Northern	H9	OL435685		
Bmm013	melanopygus	Wild	M	Red	r	43.6625278	-124.1988528	OR: Reedsport, Umpqua River Lighthouse	6/2/14	Northern	H9	OL435607	OL461855	SRR8700087
Bmm035	melanopygus	Wild	M	Red	r	43.9565444	-124.1168472	OR: Florence, Entrance of the Dunes	6/9/14	Northern	H9	OL435606	OL461856	SRR8700088
Bmm037	melanopygus	Wild	M	Red	r	44.0518139	-124.1095361	OR: Florence, Sutton Recreational Area	6/9/14	Northern	H9	OL435687	OL461858	SRR8700089
BOTM046	melanopygus	Wild	M	Black	b	41.1782051	-124.07747	CA: Patrick's Point	5/26/16	Southern	H3	OL435672		
Ferello 1	melanopygus	Wild	M	Red	r	42.102	-124.351	OR: Cape Ferello	5/25/16	Northern	H9	OL435592		

Ferello 2	melanopygus	Wild	M	Black	b	42.102	-124.351	OR: Cape Ferello	5/25/16	Northern	H9	OL435590		
Humboldt1	melanopygus	Wild	M	Black	b	40.306752	-123.903935	CA: Humboldt Redwoods State Park	5/26/16	Southern	H4	OL435675		
MLA2003	melanopygus	Wild		Black		39.55772	-123.763564	CA: Mendocino Co., 5.5 miSWestport Hwy 1	5/21/03	Southern	H4	OL435673		
OTA026	melanopygus	Wild	Q	Red		42.3169444	-122.4902778	OR: Soda Creek, Rio Rosa Ranch	4/12/16	Northern	H9	OL435655		
OTA036	melanopygus	Wild	Q	Black		42.3169444	-122.4902778	OR: Soda Creek, Rio Rosa Ranch	4/12/16	Southern	H4	OL435660		
OTA072	melanopygus	Wild	Q	Black		42.3169444	-122.4902778	OR: Soda Creek, Rio Rosa Ranch	4/12/16	Northern	H9	OL435654		
OTA081	melanopygus	Wild	Q	Red		42.3169444	-122.4902778	OR: Soda Creek, Rio Rosa Ranch	4/12/16	Northern	H9	OL435635		
OTA083	melanopygus	Wild	Q	Red		42.3169444	-122.4902778	OR: Soda Creek, Rio Rosa Ranch	4/12/16	Northern	H8	OL435640		
OTF020	melanopygus	Wild	Q	Red		42.4477778	-120.3091667	OR: NF-3630		Northern	H9	OL435588		
OTM115	melanopygus	Wild	M	Black	b	42.084	-122.651	OR: Mt. Ashland area, 5219-5311 Old Hwy 99S	5/28/16	Northern	H9	OL435650	OL461895	
OTM020	melanopygus	Wild	M	Red	r	42.147114	-122.639008	OR: Mt. Ashland area, 5219-5311 Old Hwy 99S	5/28/16	Northern	H9	OL435597		
OTM026	melanopygus	Wild	M	Black	b	42.081689	-122.706948	OR: Mt. Ashland area, 5219-5311 Old Hwy 99S	5/28/16	Southern	H4	OL435585	OL461889	
OTM043	melanopygus	Wild	M	Black	r	42.0649176	-124.3026239	OR: Rest Area N of Brookings	5/27/16	Southern	H4	OL435581	OL461890	
OTM104	melanopygus	Wild	M	Red	r	42.98	-123.75	OR: Hwy 42 W (41 ish mi to 101 N)	5/29/16	Northern	H9	OL435575		
OTM300	melanopygus	Wild	M	Red	r	42.324	-124.433	OR: Cape Sebastian	5/25/16	Northern	H9	OL435579		
OTM302	melanopygus	Wild	M	Red	r	42.102	-124.351	OR: Cape Ferello	5/25/16	Northern	H9	OL435576		
OTM303	melanopygus	Wild	M	Black	b	42.102	-124.351	OR: Cape Ferello	5/25/16	Northern	H9	OL435578		
Perpetua	melanopygus	Wild	M	Red	r	44.280985	-124.108683	OR: Cape Perpetua Visitors Center	5/24/16	Northern	H9	OL435694		
PhGG13_269_R	melanopygus	Wild	M	Red	r	42.39532	-122.28425	OR: Mt.Mcloughlin	5/13/18	Northern	H9	OL435615		
PhGG6_Site3B	melanopygus	Wild	M	Black	b	42.39532	-122.28425	OR: nr. Pacific Crest Trail, Lake of the Woods		Southern	H3	OL435677		SRR16832689
PhGG9_Site3_R	melanopygus	Wild	M	Red	r	42.39532	-122.28425	OR: nr. Pacific Crest Trail, Lake of the Woods		Northern	H9	OL435688		
Pistol	melanopygus	Wild	M	Red	r	42.276898	-124.394534	OR: Pistol River Loop	5/25/16	Northern	H9	OL435598		
QB15	melanopygus	Wild	Q	Black		38.76736	-123.5291	CA: 76 Gas Station, Gualala	1/28/17	Southern	H3	OL435666		
QB32	melanopygus	Wild	Q	Black		39.4451	-123.8064	CA: Guest House Museum, Fort Bragg	1/30/17	Southern	H3	OL435665		
QB39	melanopygus	Wild	Q	Black		39.4798	-123.7695	CA: Mendocino	1/30/17	Southern	H3	OL435663		
QB45	melanopygus	Wild	Q	Black		39.8523	-123.7414	CA: Hwy 1 roadside, Leggett	1/31/17	Southern	H3	OL435664		
QB50	melanopygus	Wild	Q	Black		40.6178	-124.2047	CA: Fernbridge Rd, Fortuna	2/1/17	Southern	H4	OL435662		
ROTM048	melanopygus	Wild	M	Red		42.100447	-124.3470295	OR: Lone Ranch Picnic Area nr. Cape Ferello	5/27/16				OL461930	
ROTM052	melanopygus	Wild	M	Red		42.2847724	-124.4070977	OR: Pistol River Loop, N. Bank Pistol River	5/25/16				OL461931	
ROTM053	melanopygus	Wild	M	Red		44.172627	-122.9500286	OR: Cape Perpetua Visitors Center	5/24/16				OL461932	
SeqNF04.42	melanopygus	Wild	M	Black	b	36.1363782	-118.5407347	CA: First turn off of 21S50	6/24/17	Southern	-			
SeqNF07.21	melanopygus	Wild	M	Black	b	36.7450869	-118.7519513	CA: off of 13S12	7/6/17	Southern	H3	OL435659		
SieNF01.27	melanopygus	Wild	M	Black	b	37.4877024	-119.6235133	CA: Goat Meadow	6/10/17	Southern	H4	OL435674		
SieNF01.28	melanopygus	Wild	M	Black	b	37.4877024	-119.6235133	CA: Goat Meadow	6/10/17					SRR8700081
StaNF01.41	melanopygus	Wild	M	Black	b	38.3450016	-119.8390321	CA: UCDSNM011758/near Dardanelle	6/18/17	Southern	H4	OL435658		
Bimac	bimaculatus	Wild	M	Black		40.730995	-77.883116	PA: Pine Grove Mills	8/1/17				OL461937	
SylB	sylvicola	Wild	M	Black		46.76846	-121.73096	WA: Mt. Rainier, Reflection Lake	7/19/15				OL461938	
SylR	sylvicola	Wild	M	Red		46.76846	-121.73096	WA: Mt. Rainier, Reflection Lake	7/19/15				OL461939	
tern	*ternarius*	Wild	W	Red		40.7705481	-77.7574485	PA: Boalsburg, Tussey Mt.	6/29/15				OL461940	
vos	*vosnesenskii*	Wild	M	Black		41.34678	-122.2414300	CA: Mt. Shasta	7/15/15				OL461942	
BIF019	vancouverensis	Wild	Q	Black		42.2567	-122.469	OR: Dead Indian Road, nr.Howard Prairie Lake	4/12/16				OL461935	
BIF209	vancouverensis	Wild	Q	Red		41.789995	-111.7734059	UT: Green Canyon Rd. Nr Logan, Utah	4/1/16				OL461936	
EPHIP66	ephippiatus	Wild	M	Black		16.7332939	-92.6722174	MEX: nr. Hirtepec Reserve	5/9/11				OL461933	
EPHIP68	ephippiatus	Wild	M	Red		16.7332939	-92.6722174	MEX: nr. Hirtepec Reserve	5/9/11				OL461934	
hunt	huntii	Wild	W	Red				UT: Logan Area	7/8/05				OL461941	
