# Supplementary material for: Mito‐nuclear discordance at a mimicry color transition zone in bumble bee Bombus melanopygus
Source: Ecol Evol. 2021 Dec 8;11(24):18151–68. doi: 10.1002/ece3.8412 (PMC8717287; doi:10.1002/ece3.8412)
Supplement: Supplementary file 4 — Table S2 [file ECE3-11-18151-s002.docx]

**Table S2:** HZAR cline parameter estimates for the best-fit model (*) based on AICc values (Color Phenotype (study samples) = II: 149.56 < I:151.14 < III: 157.26; Color Phenotype (study samples + museum samples) = II: 345.88* < I:351.67 < III: 2110.72; MtDNA Haplotype = II: 345.88* < I:351.67 < III: 2110.72; MtDNA haplotype = I: 72.27* < II: 72.87 < III: 79.48; Nuclear = I: 6.89* < II: 10.65 < III: 18.64). Center represents the location of the cline center as the distance (km) from the most southern locality, Width represents cline width, pMin represents the minimum estimated frequency at the northern side of the cline, pMax represents the maximum estimated frequency at the southern side of the cline. Values in parentheses represent two log-likelihood limits.
